# CD36 Differently Regulates Macrophage Responses to Smooth and Rough Lipopolysaccharide

**DOI:** 10.1371/journal.pone.0153558

**Published:** 2016-04-13

**Authors:** Rafał Biedroń, Angelika Peruń, Szczepan Józefowski

**Affiliations:** Department of Immunology, Jagiellonian University Medical College, Kraków, Poland; University of Leuven, Rega Institute, BELGIUM

## Abstract

Lipopolysaccharide (LPS) is the major pathogen-associated molecular pattern of Gram-negative bacterial infections, and includes smooth (S-LPS) and rough (R-LPS) chemotypes. Upon activation by LPS through CD14, TLR4/MD-2 heterodimers sequentially induce two waves of intracellular signaling for macrophage activation: the MyD88-dependent pathway from the plasma membrane and, following internalization, the TRIF-dependent pathway from endosomes. We sought to better define the role of scavenger receptors CD36 and CD204/SR-A as accessory LPS receptors that can contribute to pro-inflammatory and microbicidal activation of macrophages. We have found that CD36 differently regulates activation of mouse macrophages by S-LPS *versus* R-LPS. The ability of CD36 to substitute for CD14 in loading R-LPS, but not S-LPS onto TLR4/MD-2 allows CD14-independent macrophage responses to R-LPS. Conversely, S-LPS, but not R-LPS effectively stimulates CD14 binding to CD36, which favors S-LPS transfer from CD14 onto TLR4/MD-2 under conditions of low CD14 occupancy with S-LPS in serum-free medium. In contrast, in the presence of serum, CD36 reduces S-LPS binding to TLR4/MD-2 and the subsequent MyD88-dependent signaling, by mediating internalization of S-LPS/CD14 complexes. Additionally, CD36 positively regulates activation of TRIF-dependent signaling by both S-LPS and R-LPS, by promoting TLR4/MD-2 endocytosis. In contrast, we have found that SR-A does not function as a S-LPS receptor. Thus, by co-operating with CD14 in both R- and S-LPS loading onto TLR4/MD-2, CD36 can enhance the sensitivity of tissue-resident macrophages in detecting infections by Gram-negative bacteria. However, in later phases, following influx of serum to the infection site, the CD36-mediated negative regulation of MyD88-dependent branch of S-LPS-induced TLR4 signaling might constitute a mechanism to prevent an excessive inflammatory response, while preserving the adjuvant effect of S-LPS for adaptive immunity.

## Introduction

Macrophages and other sentinel cells detect infections with the use of pattern recognition receptors, which specifically recognize compounds produced by entire groups of related pathogens, by not by host cells, the so-called pathogen-associated molecular patterns (PAMPs). Lipopolysaccharide (LPS), a component of the outer membrane of Gram-negative bacteria, is the major PAMP signifying infections caused by these pathogens. It is recognized through the heterodimer of Toll-like receptor 4 (TLR4) with the secreted protein MD-2 [1]. LPS binding induces dimerization of TLR4/MD-2/LPS complexes, which allows dimerization of intracellular Toll/IL-1 receptor (TIR) domains of TLR4 and their binding to TIR domains present in adaptor proteins [2]. TLR4 is unique among TLRs as it engages all four adaptors involved in TLR signaling and sequentially initiates two distinct signal transduction pathways. In the plasma membrane, TLR4 induces signaling mediated by the adaptor pair TIRAP/MyD88, which leads to the early activation of NF-κB transcription factor and of mitogen-activated protein kinases and production of pro-inflammatory cytokines, such as tumor necrosis factor (TNF)-α [3]. Subsequently, TLR4/MD-2/LPS complexes undergo dynamin-dependent endocytosis through clathrin-coated pits, and within endosomes they induce the second wave of signaling, mediated by the adaptor pair TRAM/TRIF [4–6]. This TRIF-dependent pathway mediates activation of interferon-regulatory factor 3 and delayed activation of NF-κB, and is responsible for the induction of the majority of LPS-inducible genes, including type I interferons, interferon-inducible genes and some chemokines, such as RANTES [4, 7].

As the biologically active part of LPS (lipid A) is hydrophobic, its efficient binding to TLR4/MD-2 requires assistance from accessory proteins, containing hydrophobic domains which bind lipid A and prevent its thermodynamically unfavorable interactions with the polar environment. The best characterized pair of such proteins is represented by soluble LPS-binding protein (LBP) from serum and CD14, a protein attached to the plasma membrane through a glycosylphosphatidylinositol (GPI) anchor. LBP binds to LPS micelles or to surfaces of bacteria and catalyzes extraction and transfer of LPS monomers onto CD14, which, in turn, serves as the direct LPS donor for TLR4/MD-2 [8, 9]. In addition to its role in sensitizing TLR4/MD-2 to activation by very low (picomolar) concentrations of LPS, the involvement of CD14 is required for the activation of TRIF-dependent signaling, due to its role in mediating internalization of TLR4/MD-2/LPS complexes. This internalization was found independent of TLR4 signaling and to involve the Syk tyrosine kinase-dependent activation of phospholipase Cγ2 and calcium mobilization from intracellular stores [10–12].

The LPS molecule consists of phosphorylated diglucosamine substituted with 4–7 chains of long fatty acids, known as lipid A, which anchors the molecule to the membrane, to which the polysaccharide part of a varied size is attached. The polysaccharide is comprised of a more conserved core oligosaccharide, directly linked to lipid A, and the so-called O-antigen, built of units containing 3–8 glycosyl residues and repeated up to even more than 100-times. As the presence of the O-antigen in LPS is not essential for the viability of bacteria, but its synthesis is energy-demanding, in the absence of the selection pressure from the immune system some bacteria, in particular *Enterobacteriaceae*, cease to decorate their LPS with O-antigens [13]. LPS lacking the O-antigen is called rough LPS (R-LPS), because of the rough morphology of bacterial colonies producing it, as opposed to smooth appearing colonies of bacteria producing O-antigen-containing smooth LPS (S-LPS). R- and S-LPS have been often indiscriminately used in experiments. However, recent results have revealed that different LPS chemotypes may differ markedly in biological effects, likely as the result of differences in receptor usage. In particular, the involvement of CD14 is obligatory for the activation of MyD88-dependent pathways of TLR4 signaling by S-LPS, but not by R-LPS [10, 14].

In addition to LBP and CD14, several other proteins have been suggested to function as LPS uptake or accessory signaling receptors, including the class B scavenger receptor (SR)–CD36, and the class A SR–SR-A/CD204. Baranova et al. reported that CD36 is a signaling receptor for LPS, mediating the c-Jun N-terminal kinase (JNK)-dependent production of pro-inflammatory cytokines in response to both R-LPS and S-LPS [15]. These results are inconsistent with several other reports in which no effect of CD36-deficiency on macrophage responses to LPS was observed [16–18]. The role of CD36 as an autonomously signaling LPS receptor is further refuted by our observation that macrophages from C3H/HeJ mice, with a loss of function mutation in the TLR4 gene [9], fail to produce inflammatory cytokines in response to even very high concentrations of LPS (20 μg/ml), despite responding normally to CD36 ligands [19].

SR-A is able to bind lipid IVA, the tetraacylated precursor of lipid A, lipid A itself and deep rough (Re) LPS, in which the polysaccharide component is truncated to 2–3 KDO (3-deoxy-α-D-mannooctulosonic acid) residues attached to lipid A [20–22]. In contrast, there are conflicting observations regarding the role of SR-A as a receptor for natural LPS. Peiser et al. have demonstrated that LPS does not serve as the specific SR-A ligand on the surface of Gram-negative bacteria [23]. We did not observe any differences between WT and SR-A-/- macrophages in the binding of a wide range of concentrations (0.1–10 μg/ml) of biotinylated S-LPS (bS-LPS) [19]. Likewise, Drummond et al. have demonstrated that SR-A does not bind 10 μg/ml bS-LPS [24]. In contrast, SR-A has been reported to be the major macrophage receptor for uptake of fluorescent conjugates of LPS [25–27].

Acetylated low density lipoproteins, used at concentrations that block SR-A, could not alter lipid IVA or Re-LPS stimulation of TNF-α release [20], indicating that SR-A does not serve as a signaling receptor for these LPS precursors/partial structures. We have observed that SR-A deficiency has no effect on TNF-α, RANTES, IL-10 and IL-6 production, stimulated by 0.1–1 μg/ml S-LPS in murine peritoneal exudate macrophages (PEMs) [19]. Others reported unaltered S-LPS-stimulated chemokine production in SR-A-/- resident peritoneal macrophages [28]. In contrast, it has been suggested in four recent reports that SR-A participates in macrophage activation by S-LPS. However, these reports appear to be mutually conflicting [24, 27, 29, 30]. Moreover, they are difficult to reconcile with our observations that SR-A does not serve as a S-LPS receptor and that SR-A ligation with acetylated low density lipoproteins or specific mAb has no effect on LPS-stimulated TNF-α and RANTES production in macrophages [19].

In this study, we have assessed the role of CD36 and SR-A as receptors for S-LPS and for R-LPS of the Ra-type, which lacks the O-antigen, but contains the complete core structure [31]. Our results have revealed that, like CD14, CD36 plays disparate roles in regulating macrophage responses to S-LPS *versus* R-LPS. In contrast, SR-A does not seem involved in either uptake or signaling by S-LPS.

## Materials and Methods

### Reagents

Ultrapure: LPS from *Escherichia coli* K12 strain (R-LPS), LPS from *E*. *coli* 0111:B4 (S-LPS), biotinylated S-LPS (bS-LPS) and lipoteichoic acid from *Staphylococcus aureus* (LTA) were purchased from InvivoGen. Bovine serum albumin (BSA) was obtained from Roche Diagnostics, delipidated, low-endotoxin BSA, dextran sulfate (DS, MW ~500 kDa), chondroitin sulfate from bovine trachea, cytochalasin D and hydroxy-dynasore from Sigma-Aldrich and the JNK-selective inhibitor SP600125 from Tocris.

Mouse anti-mouse CD36 mAb (clone CRF D-2712) was provided by Hycult Biotech; rat anti-mouse TLR4/MD-2 complex (clones: MTS510 and Sa15-21) and rat IgG2a isotype control (RTK2758) mAb by BioLegend; rat anti-mouse TLR2 (T2.5) mAb by eBioscience; rat anti-mouse CD14 (4C1/CD14), rat anti-mouse CD11b/CR3 (M1/70), mouse IgA isotype control (M18-254) and rat IgG2b isotype control (A95-1) mAbs by BD Biosciences. With the exception of mouse IgA, the antibodies were functional grade purified (low endotoxin/no azide).

Glycolaldehyde-modified BSA (GA-BSA) was prepared, as described previously [32].

The conjugate of streptavidin with pHrodo Red (pHr-SAV) was prepared by adding 32 μl of 10.2 mM solution of pHr succinimidyl ester (Invitrogen) in dimethyl sulfoxide to 0.97 ml of streptavidin (Vector Laboratories), dissolved at 1.03 mg/ml in 0.1 M sodium bicarbonate (pH 8.4). Following 15-min incubation, unconjugated dye was separated by extensive dialysis.

### Mice

Breeding pairs of SR-A-deficient and CD36-deficient mice, both on the C57BL/6 background, as well as wild-type (WT) C57BL/6 mice were purchased from the Jackson Laboratory. Mice were housed in our facility in microisolator cages with filter tops on a 12-h light/dark cycle. This study was carried out in strict accordance with the recommendations in the Guide for the Care and Use of Laboratory Animals of the Ministry of Science and Information of Poland. The protocol was approved by the I Local Committee on the Ethics of Animal Experiments of Jagiellonian University (permit number: 83/2009). All surgery was performed under isoflurane (Abbott Laboratories) anesthesia, and all efforts were made to minimize suffering. A total of 147 mice were used in the experiments.

### Macrophages

Mice were quickly euthanized by overdose with isoflurane vapor, followed by cervical dislocation. Inflammatory peritoneal cells, elicited with 1.5 ml of 3% Thioglycollate (Difco Laboratories), injected *i*.*p*. 4 days earlier, were washed out with phosphate-buffered saline (PBS). The cells were re-suspended at 0.8 × 10^6^ in FCS-RPMI [RPMI 1640 medium with HEPES, supplemented with 10% fetal calf serum (FCS), 2 mM L-glutamine (Cytogen), and 0.04 mg/ml gentamycin (KRKA)] and, unless otherwise indicated, plated at 1.6 × 10^5^/well in 96-well tissue culture-treated plates. After overnight incubation, non-adherent cells were removed by washing and adherent macrophages (PEM) were used in the experiments described below.

### Binding and uptake of bS-LPS

#### Binding

Adherent PEMs were incubated for 1 h on ice with bS-LPS in FCS-RPMI, as described previously [19]. Subsequently, the cells were washed 3 times with 0.5% BSA in PBS (BSA-PBS) and incubated for another 1 h with 5 μg/ml horseradish peroxidase-streptavidin conjugate (HRP-SAV, Vector Laboratories) in BSA-PBS. Following extensive washing, the enzymatic reaction for peroxidase was performed with the use of TMB Substrate Reagent Set (BD Biosciences) as the substrate. The absorbance of the product was measured in a plate reader (PowerWave, Bio-Tek Instruments). Alternatively, PEMs were incubated with 5 μg/ml phycoerythrin-SAV conjugate (eBioscience), instead of HRP-SAV, and cell-associated fluorescence was quantified in a fluorescence plate reader (Infinite M200 PRO, Tecan).

#### Uptake

PEMs were incubated for 1 h on ice with 0.2 or 2 μg/ml bS-LPS, as described above. Following washing, 60 μl/well of 10 μg/ml pHr-SAV in BSA-PBS was added for 50-min incubation on ice. Subsequently, the cells were washed 3 times, 0.14 ml of FCS-RPMI was added and the plates were placed for 100 min in a cell culture incubator. Finally, the wells were washed once, filled with 0.1 ml PBS which pH was adjusted to 9.0 with 25 mM HEPES, and the fluorescence of PEMs was quantified in the fluorescence plate reader.

### Binding assays with recombinant receptors

#### Preparation of coated plates

Ninety six-well, half area ELISA plates (Corning Inc.) were coated by overnight incubation at 4–8°C with 45 μl of PBS containing 5 μg/ml of recombinant mouse receptors (R&D Systems), 20 μg/ml GA-BSA or 50 μg/ml LPS or LTA. The plates were washed twice with 0.05% Tween 20 in PBS or BSA-PBS (LPS- or LTA-coated plates), blocked for 1 h at room temperature with 150 μl/well of 10% FCS in PBS (FCS-PBS) or 2% BSA in PBS (LPS- or LTA-coated plates), washed again and used in the experiments described below.

Levels of adsorption of receptors to plates were compared by a direct ELISA method, with the use of 2.5 μg/ml of HRP-conjugated F(ab’)_2_ fragments of goat antibodies specific for Fc fragments of human IgG (HRP-anti-Fc, Rockland), as recombinant receptors we used were tagged with the Fc fragment of human IgG1.

#### Binding of bS-LPS

Different concentrations of bS-LPS in FCS-PBS or BSA-PBS were added in 45 μl to plates coated with recombinant receptors for 80-min (rCD36) or 1-h (rCD14, rLBP) incubation at 37°C. Subsequently, the plates were washed 4 times with BSA-PBS and incubated for 30 min at 37°C with 4 μg/ml of HRP-SAV in BSA-PBS. Following 6-times washing, the enzymatic reaction for peroxidase was performed. In parallel, non-specific binding to wells not coated with receptors was determined for each bS-LPS concentration and subtracted from the total binding to receptor-coated wells to obtain the receptor-specific binding.

#### bS-LPS transfer between receptors

rCD36 adsorbed to ELISA plates was loaded with bS-LPS by 80-min incubation at 37°C with 45 μl of 500 ng/ml bS-LPS in BSA-PBS. Similarly, plate-adsorbed rCD14 and rLBP were loaded with 250 ng/ml bS-LPS in, respectively, FCS-PBS and BSA-PBS. After washing 4 times with BSA-PBS, 90 μl/well of 5 μg/ml recombinant receptors or other potential LPS acceptors was added for 1-h incubation at 37°C. Following washing, bS-LPS that remained bound to immobilized receptors was detected with HRP-SAV, as described above.

#### Direct binding between receptors

Plate-adsorbed receptors were incubated for 90 min at 37°C with different concentrations of soluble receptors, with or without 1 μg/ml LPS, in 60 μl FCS-PBS. Subsequently, the plates were washed 4-times with BSA-PBS. Bound rCD36 was detected by 1-h incubation with 2 μg/ml of polyclonal goat anti-mouse CD36 Ab (R&D Systems), followed by 1-h incubation with 4 μg/ml of HRP-conjugated F(ab’)_2_ fragments of donkey anti-goat IgG Ab (Rockland) in BSA-PBS. Bound rLBP was detected with 1:400 dilution of HRP-conjugated mouse monoclonal IgG1 anti-6×Histidine (R&D Systems).

#### Competition experiments with rCD36

rCD36 (2 μg/ml) was pre-incubated for 20 min at 37°C with competitors (100 μg/ml DS or CS, 5 μg/ml anti-CD36 or control IgA mAb, 200 μg/ml LTA or LPS), before being added to GA-BSA-coated plates for 60-min incubation at room temperature. Subsequently, plates were washed 3 times with Tween/PBS and bound rCD36 was detected by 50-min incubation with 2 μg/ml of HRP-anti-Fc in BSA-PBS. Binding of rCD36 to LPS- or LTA-coated plates was assessed in a similar manner, except that BSA-PBS, instead of Tween/PBS was used as the washing buffer.

### Binding and uptake of fluorescently-labeled S-LPS

#### Fluorescent LPS

A conjugate of oxidized S-LPS with the Alexa Fluor 647 fluorescent dye (AF-LPS) was prepared as follows. S-LPS was suspended at 1 mg/ml in 0.5 ml of 0.1 M sodium acetate (pH 5.0) and incubated with 1 mM sodium metaperiodate (Sigma-Aldrich) for 30 min on ice [33]. The reaction was stopped by adding 4 μl of glycerol and oxidized LPS was separated from other reagents by extensive, 24-h dialysis against PBS at 4°C (6–8 kDa cutoff). Fifty μl of Alexa Fluor 647 hydrazide (Invitrogen), dissolved at 50 mM in dimethyl sulfoxide, was added to 0.5 ml of LPS for 2-h incubation at room temperature. Unreacted dye was separated by dialysis. The estimated degree of labeling in this AF-LPS preparation was ~0.47:1 (AF:S-LPS).

To prepare a conjugate of native S-LPS with BODIPY FL (BO-LPS), S-LPS was suspended at 2 mg/ml in 0.25 ml of PBS, sonicated and mixed with 25 μl of 50 mM BODIPY FL hydrazide (Invitrogen) in dimethyl sulfoxide. After 40-min incubation at 37°C, the mixture was sonicated again, 0.2 ml of 0.2 M NaHCO_3,_ (pH 8.7) and another 25 μl of BODIPY FL hydrazide were added and the incubation was continued for further 1 h. The sample was then microfuged and the supernatant dialyzed. The estimated degree of labeling in BO-LPS was ~0.13:1.

#### Uptake

PEMs were incubated for 70 min at 37°C in 0.16 ml FCS-RPMI, containing the indicated concentrations of fluorescently-labeled LPS preparations. Then, the cells were washed 3-times with PBS and their fluorescence was measured in the fluorescence plate reader or by flow cytometry, following detachment with lidocaine/EDTA [34].

#### Binding

In the binding experiments, PEMs were metabolically-poisoned by 30-min incubation at 37°C with 80 μl of PBS with Ca/Mg, containing 1 mM NaN_3_, 2 mM NaF and 50 mM 2-deoxyglucose (Sigma-Aldrich), in order to prevent internalization of bound ligands. Subsequently, 80-μl volumes of 20% FCS in Ca/Mg-PBS, containing double-concentrated solutions of fluorescent ligands, were added and the incubation was continued for 70 min. The rest of the procedure was the same as in the uptake experiments.

### Binding of rCD14 to PEMs

PEMs were pre-incubated for 40 min at 37°C with 20% mouse serum in FCS-RPMI. Subsequently, the medium was replaced with 30 μl of double-concentrated solutions of receptor-blocking mAb in FCS-RPMI for 30 min pre-incubation at room temperature, followed by addition of 30 μl of double-concentrated solution of rCD14 (the final concentration 5 μg/ml), with or without LPS, and incubation at 37°C for 50 min. The cells were washed 4-times with ice-cold BSA-PBS and incubated for 50 min on ice with 2.5 μg/ml of HRP-anti-Fc. After extensive washing, the enzymatic reaction for peroxidase was performed.

### Stimulation of cytokine production

Peritoneal exudate cells were plated at 1.12 × 10^5^/well. PEMs were incubated for 1 h on ice with 1 μg/ml LPS or for 40 min at 37°C with 200 ng/ml LPS in 0.14 ml FCS-RPMI or serum-free medium (BSA-RPMI), containing 0.5% low-endotoxin BSA instead of FCS. Subsequently, unbound LPS was removed by washing 2-times with PBS, 0.14 ml of fresh FCS-RPMI was added and the incubation was continued for 3 h in a cell culture incubator. To block CD14 or TLR4/MD-2, the cells were pre-incubated for 30 min at room temperature with 70 μl of double-concentrated solutions of mAb (40 μg/ml), before the same volume of LPS-containing medium was added. Pre-incubated with pharmacological inhibitors was performed at 37°C for 30 min. Cytokine concentrations in culture supernatants were determined by ELISA, as described previously [19].

Monomeric S-LPS/rCD14 complexes were prepared by an overnight incubation at 37°C of 2 μg/ml S-LPS with 50 μg/ml rCD14 (R&D Systems) in BSA-PBS [35].

### Assessment of LPS binding to TLR4/MD-2

For assessing the degree of TLR4/MD-2 occupancy by LPS, we exploited the ability of LPS to inhibit binding of clone MTS510 anti-TLR4/MD-2 mAb to the receptor complex [36] in cellular ELISA [37]. PEMs were incubated for 40 min at 37°C with 200 ng/ml of S-LPS or R-LPS in 0.2 ml FCS-RPMI or BSA-RPMI. The cells were washed once with PBS and incubated for 20 min on ice with 40 μl of 40% mouse serum in FCS-RPMI. Subsequently, 40 μl of double-concentrated solutions of MTS510 or control rat IgG2a mAb was added (the final concentration 5 μg/ml) and the incubation was continued for 1 h. Unbound mAb were removed by 3-times washing with ice-cold BSA-PBS and PEMs were subjected to another 50-min incubation on ice with 5 μg/ml of HRP-conjugated F(ab’)_2_ fragments of goat Ab anti-rat IgG, pre-adsorbed with mouse serum proteins (Rockland) in 70 μl of FCS-RPMI. The wells were washed 6-times and the enzymatic reaction for peroxidase was performed, as described above. The specific binding was calculated by subtracting binding of control rat IgG2a from the total binding of MTS510 mAb. In parallel, the effect of each treatment on cell surface expression of TLR4/MD-2 was determined with the use of clone Sa15-21 mAb, which binding to TLR4/MD-2 is unaffected by LPS [36], and used for the normalization of data.

### Data analysis

The data were analyzed with the Student’s t-test, for single comparisons, or ANOVA, for multiple comparisons, with the Tukey-Kramer post-test used to compare all pairs of groups, and the Dunnett’s test to compare all other groups to the control group (GraphPad Prism software). The p values < 0.05 were assumed to denote statistically significant difference. The GraphPad Prism program was also used to calculate receptor binding parameters by non-linear regression curve fitting.

## Results

### Binding of S-LPS to PEMs is mediated by CD14, but not by CD36 or SR-A

**Fig 1:**
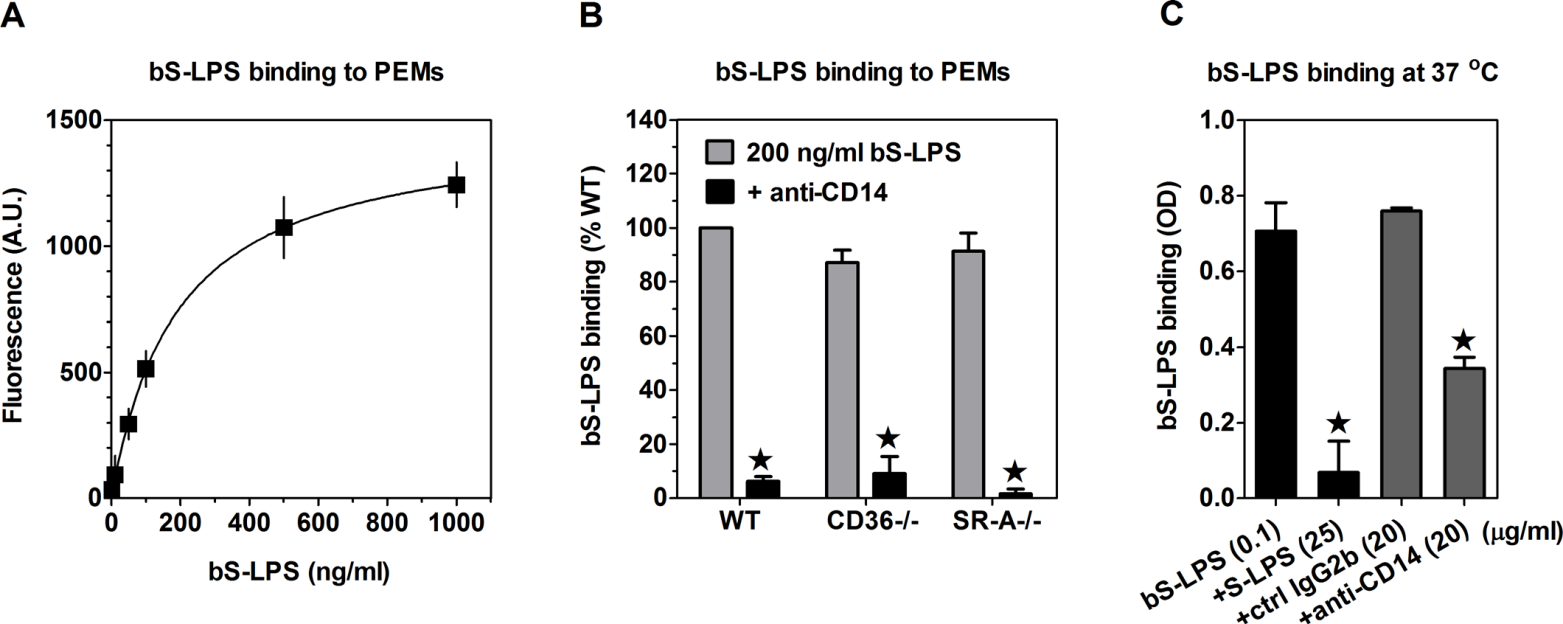
Binding of S-LPS to PEMs in serum-containing medium is mediated by CD14. (A) bS-LPS binds dose-dependently to WT PEMs. (B) WT, CD36-/- and SR-A-/- PEMs exhibit similar binding of bS-LPS, which is fully blocked by anti-CD14 mAb. (C) Binding of bS-LPS at 37°C to metabolically poisoned PEMs is inhibited by anti-CD14 mAb and by an excess of unlabeled S-LPS. Graphs show averages +SEM from 3 independent experiments (B) or results of single experiments, performed in 4 replicates, each representative of 3 such experiments performed (A, C). *, significant inhibition of bS-LPS binding (p<0.05), according to Student’s t-test (B) or ANOVA, followed by the Dunnett’s post-test (C); A.U., arbitrary units.

PEMs exhibited dose-dependent, saturable binding of bS-LPS (K_D_ ~190 ng/ml) during 1-h incubation on ice in serum-containing medium (Fig 1A). This binding was prevented by blocking anti-CD14 mAb, but was not significantly affected by CD36 or SR-A deficiency (Fig 1B). Likewise, bS-LPS binding to metabolically poisoned PEMs at 37°C was strongly inhibited by anti-CD14 mAb (Fig 1C), but unaffected by CD36 or SR-A deficiency (S1A Fig). However, anti-CD14 mAb consistently produced stronger inhibition of bS-LPS binding on ice than at 37°C. Binding of bS-LPS to PEMs, both at 37°C (Fig 1C) and on ice (S1B Fig), was also blocked by 200-250-fold excess of unlabeled S-LPS, confirming that S-LPS itself rather than the attached biotin is responsible for bS-LPS binding to PEMs.

In serum-free medium (BSA-PBS), bS-LPS bound to rCD14 with a K_D_ of 113 ± 20.9 ng/ml (mean ± SEM from 6 experiments, S2A Fig). The inclusion of serum produced a ~3-fold increase of the maximal bS-LPS binding to rCD14 (S2A Fig), but the affinity of binding did not significantly change (K_D_ = 87 ± 16.0 ng/ml, N = 4). Lower bS-LPS binding to rCD14 in serum-free, as compared to serum-containing medium during 1-h incubation was caused by much slower binding kinetics (S2B Fig). LBP seems to be the serum component responsible for the acceleration of bS-LPS binding to rCD14, because the addition of 200 ng/ml rLBP to BSA-PBS increased the 1-h binding to levels even exceeding those observed in the presence of serum (S2C Fig). No further increase of the binding was produced by higher concentrations of rLBP (S2E Fig).

**Fig 2:**
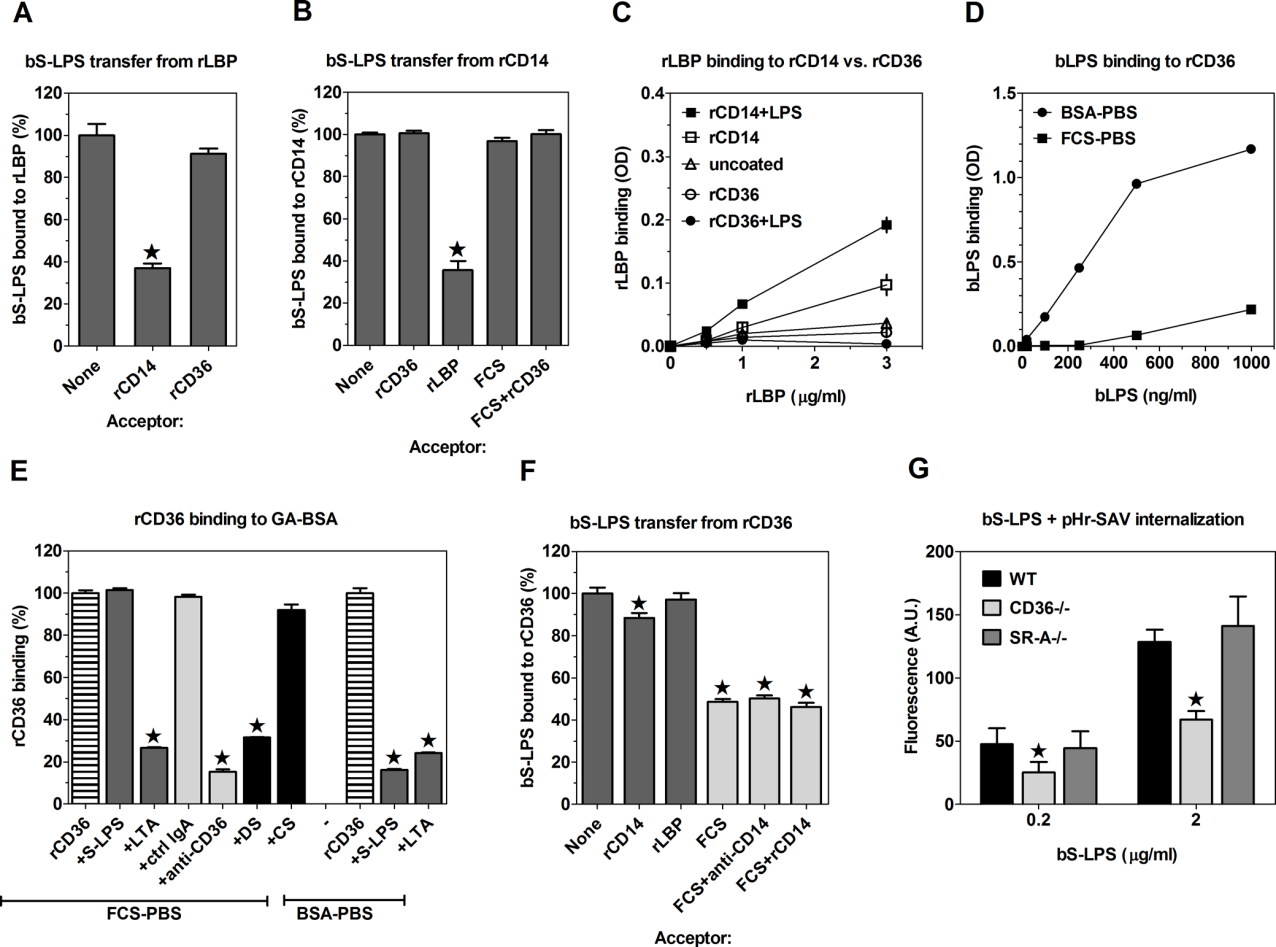
The role of CD36 as a S-LPS receptor. (A) Incubation with 5 μg/ml rCD14, but not with rCD36 partially removes bS-LPS from rLBP. (B) rLBP, but not rCD36 or serum depletes rCD14 of bound bS-LPS. (C) rLBP binds very weakly to plate-adsorbed rCD14, but not to rCD36 and this binding is only slightly increased by 1 μg/ml S-LPS. (D) bS-LPS binds much more strongly to plate-adsorbed rCD36 in BSA-PBS than in FCS-PBS. (E) rCD36 binding to plate-adsorbed GA-BSA is inhibited by 0.2 mg/ml LTA, 0.1 mg/ml DS and anti-CD36 mAb, whereas 0.2 mg/ml S-LPS inhibits rCD36 binding only in the absence of serum. Chondroitin sulfate (CS) and control IgA have no effect on the binding. (F) A large portion of bS-LPS bound to rCD36 is removed by serum component(s) distinct from CD14 or LBP. (G) Relative to WT controls, internalization of bS-LPS/pHr-SAV complexes is significantly decreased in CD36-/-, but not SR-A-/- PEMs. Graphs show means ± SEM of 3–5 replicates, obtained in single experiments, each representative of at least 3 similar experiments performed (A-F) or averages + SEM from 4 independent experiments (G). Data were analyzed with the regular (A, B, E and F) or repeated measures (G) ANOVA, followed by the Dunnett’s post-test. *, p < 0.05; OD, optical density.

In experiments aimed to elucidate the mechanism of the enhancing effect of rLBP on bS-LPS binding to rCD14, we have found that rLBP is able to bind bS-LPS, but with slightly lower affinity (K_D_ = 189 ± 16.9 ng/ml, N = 3; S2D Fig) than rCD14. rLBP-bound bS-LPS may be then transferred to rCD14 (Fig 2A). Interestingly, this transfer is reversible, as indicated by the decrease of the amount of bS-LPS bound to rCD14 caused by post-incubation with rLBP (Fig 2B). There was a weak binding between rLBP and rCD14, which was only significant at rLBP concentrations higher than that sufficient to produce the maximal acceleration of bS-LPS binding to rCD14 (Fig 2C). This low-affinity binding may reflect transient association between rCD14 and rLBP, required for the transfer of S-LPS between these proteins.

rCD36 exhibited dose-dependent binding of bS-LPS, but binding of physiologically relevant concentrations of bS-LPS (<500 ng/ml) was only observed in BSA-PBS, but not in FCS-PBS (Fig 2D). Binding of bS-LPS to rCD36 was inhibited in ~74% by anti-CD36 mAb (S3A Fig). However, the affinity of bS-LPS binding to rCD36 (K_D_ = 837 ± 18.8 ng/ml, N = 3) was ~7.4-times lower than that to rCD14. Also the value of maximal binding was ~2.4-fold lower in the case of rCD36. Consequently, even in BSA-PBS, binding of bS-LPS to rCD36 was very low relative to that to rCD14 (S2A Fig); also under serum free conditions there was no difference in bS-LPS binding at 37°C between metabolically-poisoned WT and CD36-/- PEMs (S1A Fig). The difference in bS-LPS binding to rCD36 *versus* rCD14 was not caused by different amounts of receptors adsorbed to plates (S3B Fig).

We also assessed the role of CD36 as a receptor for S-LPS and LTA in competition experiments with soluble rCD36. In the presence of serum, binding of rCD36 to plate-adsorbed GA-BSA was strongly inhibited by 0.2 mg/ml LTA, whereas the same concentration of S-LPS had no effect (Fig 2E). In contrast, in serum-free medium S-LPS inhibited rCD36 binding to GA-BSA to a similar extent as LTA. rCD36 binding to GA-BSA was also inhibited by anti-CD36 mAb and DS, but not by chondroitin sulfate, a control polyanion which does not bind to SRs (Fig 2E).

Incubation of bS-LPS/rCD36 complexes with serum decreased the amount of bS-LPS bound to rCD36 by ~50% (Fig 2F), which may explain the apparent lack of bS-LPS binding to rCD36 in serum-containing medium. However, our results do not support the role of LBP or CD14 as the serum component mediating bS-LPS depletion from rCD36, as recombined receptors or blocking mAb had little or no effect on the amount of bS-LPS bound to rCD36 (Fig 2F).

### CD36, but not SR-A participates in S-LPS uptake by PEMs

In order to assess the role of SR-A and CD36 in the uptake of S-LPS by PEMs, we examined effects of receptor deficiencies on the uptake of Alexa Fluor 647-labeled S-LPS (AF-LPS). Relative to WT controls, in both SR-A-/- and CD36-/- PEMs uptake of 1–5 μg/ml AF-LPS was decreased by ~21–25% (S4A Fig). Consistent with the major role of SRs, the uptake of AF-LPS was reduced in ~62% by 0.1 mg/ml DS (S4B Fig). We then assessed AF-LPS binding to metabolically-poisoned PEMs. As shown in S4C Fig, pre-incubation with NaN_3_, NaF and 2-deoxyglucose blocked internalization into acidic endosomes of pHr-labeled, HOCl-oxidized ovalbumin (pHr-OVA-Cl), a ligand of both SR-A and CD36 [34], which confirms the effectiveness of this treatment in blocking endocytosis. The binding of AF-LPS to metabolically-poisoned PEMs was several-fold lower than its uptake, indicating that AF-LPS becomes endocytosed (S4B Fig). However, unlike bS-LPS binding, both the uptake and the binding of AF-LPS were not saturable and increased linearly up to 5 μg/ml AF-LPS (S4D Fig). Moreover, neither the uptake nor the binding of AF-LPS were inhibited by as high as 250-fold excess of unlabeled S-LPS (S4B Fig). These results indicate that AF-LPS binding to PEMs is mediated by the attached fluorochrome (AF) rather than by S-LPS itself.

A shared feature of SR ligands is their net negative charge. A strong negative charge is conferred on the Alexa Fluor 647 molecule by 4 sulfite groups. We therefore prepared a weakly-labeled conjugate of native, unoxidized S-LPS with an uncharged fluorescence dye–BODIPY FL (BO-LPS) and assessed its uptake by PEMs. In CD36-/-, but not in SR-A-/- PEMs uptake of BO-LPS was significantly decreased (S4E Fig). However, 200-fold access of unlabeled S-LPS produced only partial, ~32% inhibition of BO-LPS uptake (S4F Fig). These results indicate that BO-LPS uptake by PEMs is mediated by both S-LPS itself and by the attached FL BODIPY dye.

The above results question the usefulness of fluorescent LPS conjugates and suggest that the observed in the previous studies binding of such conjugates to SR-A [25–27] might be an artifact caused by the attached, negatively-charged fluorochromes. We therefore resorted to studying uptake of bS-LPS, which, as shown above (Fig 1C), retains binding specificity of native S-LPS. In these experiments, bS-LPS bound to CD14 on the surface of PEMs was labeled with pHr-SAV and then the cells were incubated for 100 min at 37°C, in order to enable internalization of bS-LPS/pHr-SAV complexes. As shown in Fig 2G, in CD36-/-, but not in SR-A-/- PEMs internalization of bS-LPS into acidic endosomes was strongly reduced (by ~47%).

### Binding of S-LPS to CD14 induces its association with CD36, not accompanied by S-LPS transfer

The results obtained so far indicate that although S-LPS binding to PEMs in serum-containing medium is mediated by CD14 but not CD36, CD36 co-operates with CD14 in S-LPS endocytosis. Two mechanisms of this cooperation seemed possible to us. First, CD14 may transfer S-LPS onto CD36, which then mediates S-LPS internalization. Alternatively, S-LPS binding to CD14 induces its association with CD36 and both receptors are internalized together. The fact that S-LPS binds to CD14 with much higher affinity than to CD36 makes the transfer of S-LPS from CD14 to CD36 thermodynamically unfavorable. Consistently, we observed no transfer of bS-LPS from rCD14 to rCD36 (Fig 2B).

**Fig 3:**
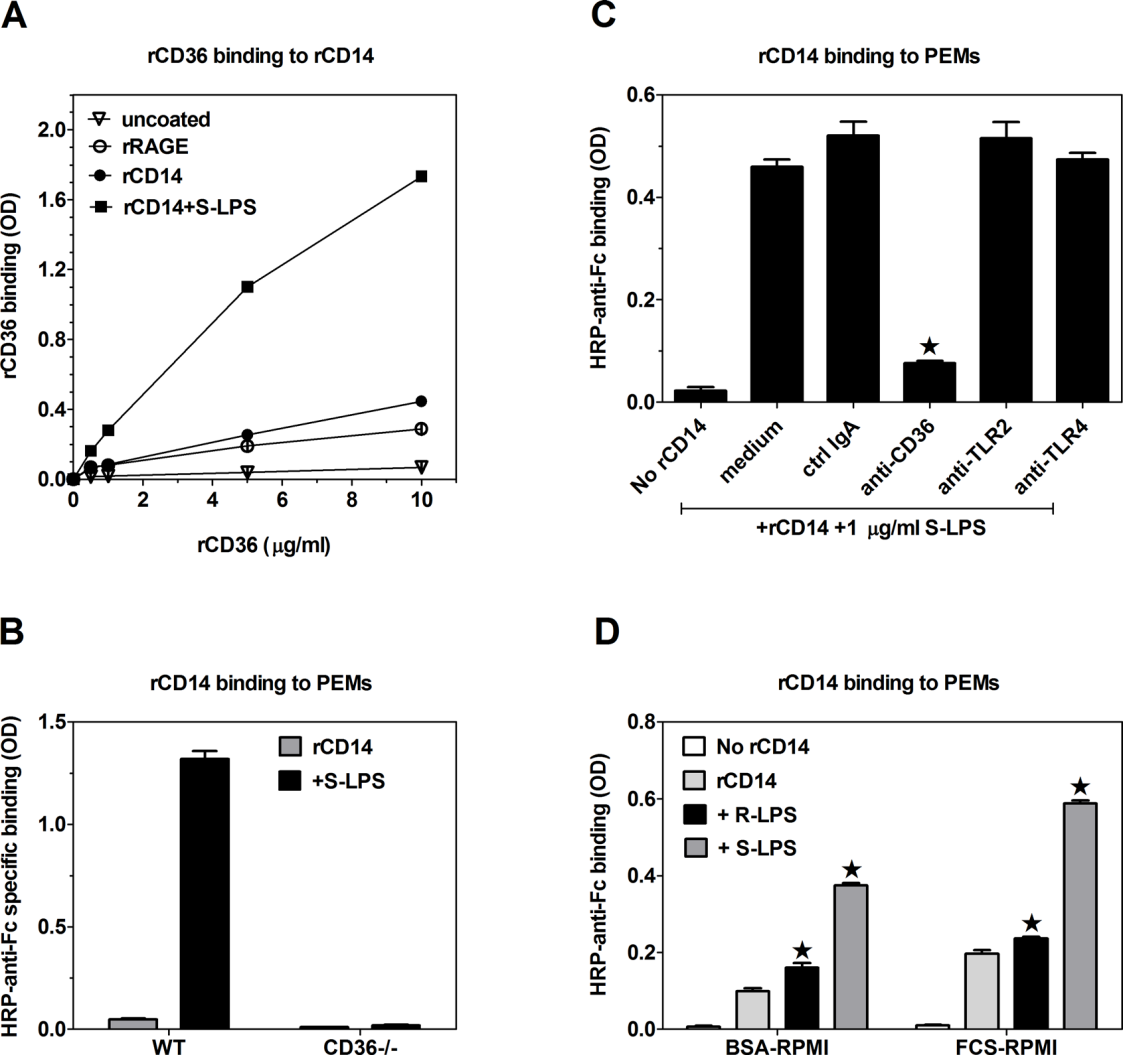
S-LPS stimulates association of CD14 with CD36. (A) S-LPS at 1 μg/ml enhances binding of rCD36 to plate-adsorbed rCD14. (B) CD36-/- PEMs exhibit neither basal nor 1 μg/ml S-LPS-stimulated rCD14 binding. (C) S-LPS-stimulated binding of rCD14 to PEMs is blocked by anti-CD36 mAb, but not by anti-TLR2 or anti-TLR4/MD-2 mAb. (D) During 40 min co-incubation at 37°C, 200 ng/ml S-LPS stimulates much stronger rCD14 binding to PEMs than the same concentration of R-LPS. Graphs show means ± SEM of 3–4 replicates, obtained in single experiments, each representative of at least 3 similar experiments performed. Data on graphs C and D were analyzed with ANOVA, followed by the Dunnett’s post-test. *, p < 0.05; HRP-anti-Fc, HRP-conjugated F(ab’)_2_ fragments of goat antibodies specific for Fc fragments of human IgG.

We started testing the second model, assuming S-LPS-induced association of CD14 with CD36, by examining the ability of rCD14 to bind to rCD36 and the influence of S-LPS on this interaction. As a negative control we studied association of rCD36 with rRAGE, which, like rCD14 and rCD36, was tagged with the Fc fragment of human IgG1. Only very weak binding of rCD36 to plate-adsorbed rRAGE could be detected, which may be considered as non-specific binding (Fig 3A). In comparison to rRAGE-rCD36 interactions, rCD36 exhibited slightly increased binding to rCD14, but only at rCD36 concentrations ≥ 5 μg/ml. However, the inclusion of 1 μg/ml S-LPS stimulated strong binding between rCD36 and rCD14, which was already evident at the lowest tested concentration of rCD36 (0.5 μg/ml) (Fig 3A). We then assessed interactions of rCD14 with endogenous receptors expressed on the surface of PEMs. In the absence of LPS, rCD14 exhibited only low-level binding to PEMs (Fig 3B). The inclusion of S-LPS produced >25-fold increase of rCD14 binding to PEMs. Even higher (~2-fold) rCD14 binding to PEMs was observed during incubation on ice (S1C Fig), suggesting that at 37°C a portion of PEM-bound rCD14 may undergo internalization. The S-LPS-stimulated rCD14 binding to PEMs was selectively and almost completely inhibited by anti-CD36 mAb, whereas blocking mAb against TLR2 or TLR4/MD-2 were ineffective (Fig 3C). The exclusive role of CD36 in mediating rCD14 binding to PEMs was confirmed in experiments with CD36-deficient PEMs, which exhibited neither basal nor S-LPS-stimulated rCD14 binding (Fig 3B).

### In the presence of serum, CD36 reduces S-LPS-induced, MyD88-dependent TLR4 signaling by competing with TLR4/MD-2 for S-LPS/CD14 complexes

**Fig 4:**
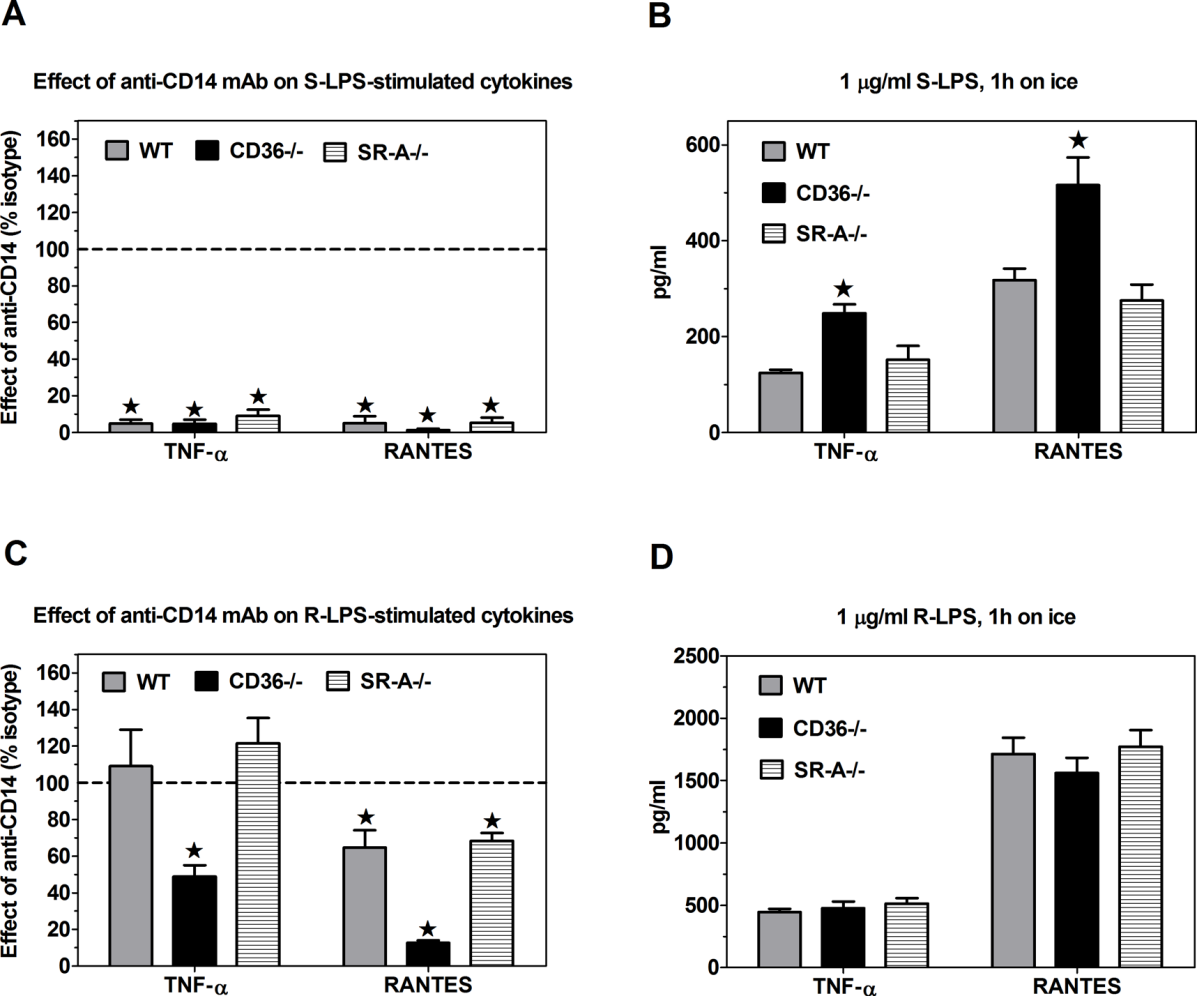
**Effects of CD36 or SR-A deficiency and of anti-CD14 mAb on TNF-α and RANTES production, stimulated by 1-h incubation on ice with 1** μ**g/ml S-LPS (A-B) or R-LPS (C-D).** (A) Anti-CD14 mAb blocks both TNF-α and RANTES production, stimulated by S-LPS. (B) S-LPS stimulates significantly higher cytokine production in CD36-/- than in WT or SR-A-/- PEMs. (C) R-LPS-stimulated cytokine production is more strongly inhibited by anti-CD14 mAb in CD36-/- than in WT or SR-A-/- PEMs. (D) R-LPS stimulates similar cytokine production in WT, CD36-/- and SR-A-/- PEMs. Graphs show means +SEM from 4–6 independent experiments, each performed in 4 replicates. Data on graphs A and C were analyzed with one-sample t-test, and on graphs B and D with ANOVA, followed by the Dunnett’s test. *, p < 0.05.

In order to assess functional consequences of S-LPS/CD14 binding to CD36, WT and knockout PEMs were incubated with 1 μg/ml S-LPS for 1 h on ice. Subsequently, unbound S-LPS was washed out and the cells were transferred to 37°C for 3-h incubation. The activation of MyD88-dependent and TRIF-dependent signaling pathways was monitored by measuring levels of TNF-α and RANTES, respectively, in culture supernatants. Production of both TNF-α and RANTES was strictly CD14-dependent (Fig 4A), which is consistent with the observation that under these conditions S-LPS binding to PEMs is selectively mediated by CD14 (Fig 1B). In comparison to WT controls, in CD36-/-, but not in SR-A-/- PEMs S-LPS stimulated significantly higher production of both cytokines (Fig 4B). These results indicate that CD36-mediated sequestration of S-LPS/CD14 complexes partially prevents TLR4/MD-2 activation by S-LPS.

**Fig 5:**
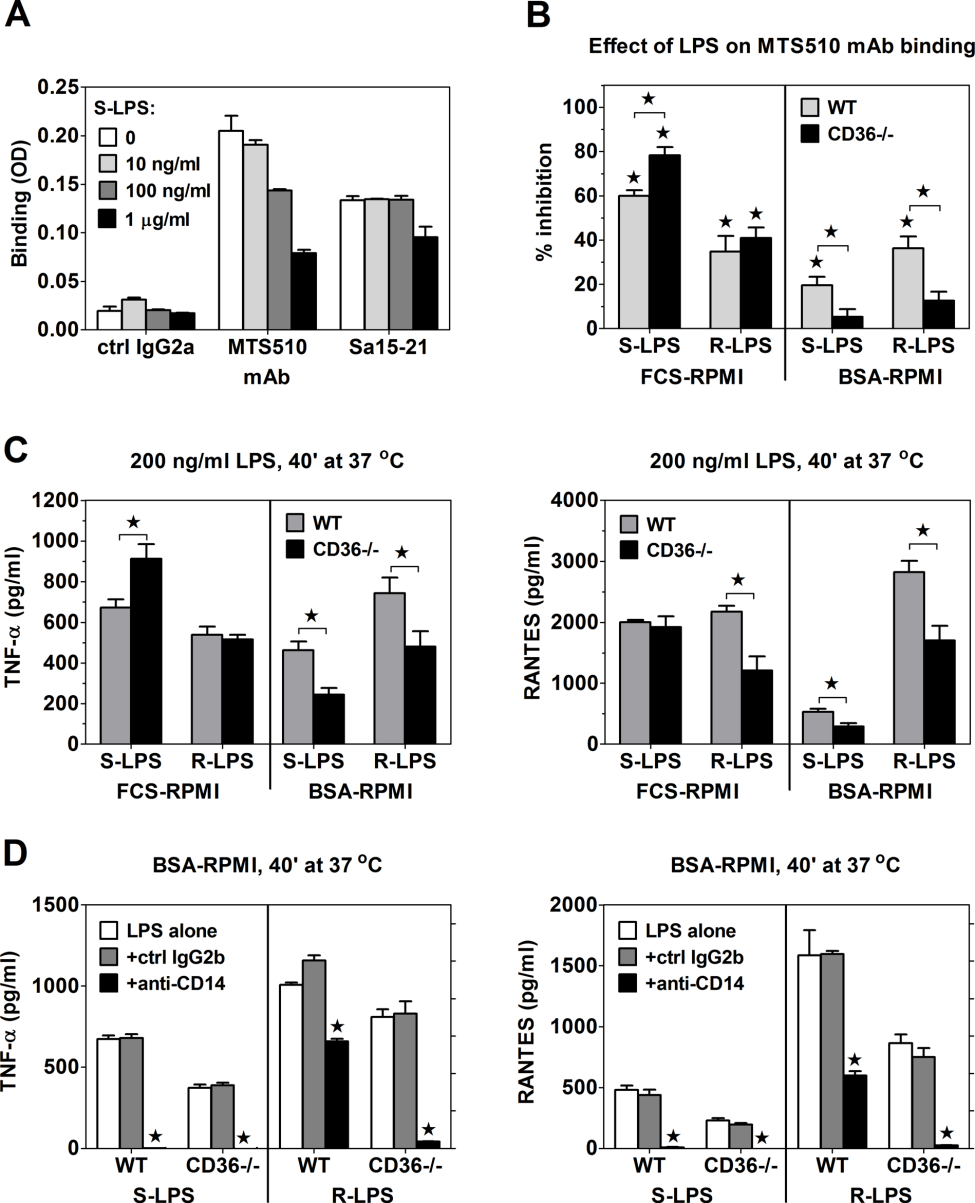
Relative roles played by CD36 and CD14 in LPS loading onto TLR4/MD-2 and in LPS-stimulated cytokine production depend on the LPS chemotype and the presence of serum. (A) Binding of mAbs to PEMs pre-incubated with the indicated concentrations of S-LPS. (B) Forty-min pre-incubation at 37°C with 200 ng/ml S-LPS, but not with R-LPS in FCS-RPMI more strongly inhibits MTS510 mAb binding to TLR4/MD-2 on CD36-/- than on WT PEMs. In contrast, in BSA-RPMI both S-LPS and R-LPS produce significant inhibition of MTS510 mAb binding only in WT PEMs. (C) Forty-min stimulation in FCS-RPMI with 200 ng/ml S-LPS, but not with R-LPS induces significantly higher TNF-α production in WT than in CD36-/- PEMs (left panel). In contrast, R-LPS, but not S-LPS stimulates significantly lower RANTES production in CD36-/- PEMs (right panel). In BSA-RPMI, CD36-/- PEMs exhibit severe impairment of cytokine production in response to both S-LPS and R-LPS. (D) In both WT and CD36-/- PEMs cytokine production stimulated by 40-min incubation with 200 ng/ml S-LPS in serum-free medium is blocked completely by anti-CD14 mAb. Anti-CD14 mAb also blocks cytokine production stimulated by R-LPS in CD36-/- PEMs, whereas it exerts only partial inhibition in WT PEMs. Data shown on graphs A and D are means +SEM of triplicates, obtained in single experiments, which were performed 2 (A) or 3 (D) times with similar results. Graphs B-C show means +SEM from 4–6 independent experiments, each performed in 4 replicates. *, significant inhibition or difference between WT and CD36-/- PEMs (p < 0.05 in Student’s t-test).

Higher binding of S-LPS to TLR4/MD-2 on CD36-/- as compared to WT PEMs has been confirmed in experiments with clone MTS510 anti-TLR4/MD-2 mAb, which does not bind to LPS-occupied TLR4/MD-2 [36]. Similarly as in our previous study on J774 macrophage-like cells [37], only concentrations of S-LPS higher than 100 ng/ml caused down regulation of cell surface TLR4/MD-2 expression, assessed with clone Sa15-21 anti-TLR4/MD-2 mAb (Fig 5A). In contrast, binding of clone MTS510 mAb was dose-dependently inhibited by S-LPS. When incubated with PEMs for 40 min at 37°C, 200 ng/ml S-LPS produced significantly stronger inhibition of MTS-510 mAb binding to TLR4/MD-2 on CD36-/- than on WT PEMs (Fig 5B), confirming that CD36-mediated internalization of S-LPS/CD14 complexes partially prevents S-LPS transfer from CD14 onto TLR4/MD-2. The higher TLR4/MD-2 occupancy by S-LPS in CD36-/-, as compared to WT PEMs was paralleled by significantly higher TNF-α production (Fig 5C, left panel).

PEMs expressed ~10-times more CD14 than TLR4/MD-2 on their surface (S5A Fig). The expression of both receptors was not altered in CD36-/- relative to WT PEMs (S5B Fig), ruling out the difference in expression levels of these receptors as a possible cause of the observed differences in responsiveness to S-LPS. We have reported previously that also SR-A-/- PEMs express similar levels of CD14 and TLR4/MD-2 as WT PEMs [19]. Consistent with our previous observations [34], PEMs expressed very high levels of CD36, ~4-fold higher than CD14, and the expression of this receptor was not affected by SR-A deficiency (S5A Fig).

### In serum-free medium, binding of S-LPS/CD14 complexes to CD36 promotes S-LPS transfer from CD14 onto TLR4/MD-2

As S-LPS binds much more strongly to rCD36 in serum-free than in serum-containing medium, we assessed the role of CD36 in the regulation of macrophage activation by S-LPS under the former conditions. In serum-free medium (BSA-RPMI), binding of S-LPS to TLR4/MD-2 expressed on the surface of CD36-deficient PEMs was severely impaired (Fig 5B) and, consequently, CD36-/- PEMs produced much less of both TNF-α and RANTES upon stimulation with S-LPS than WT PEMs (Fig 5C). The observation that rCD36 has no effect on bS-LPS binding to rCD14 (S3C Fig) indicates that CD36 does not indirectly facilitate S-LPS loading onto TLR4/MD-2 by mediating disaggregation of S-LPS micelles and transfer of monomers to CD14, in a manner analogous to LBP. Also the possibility of the direct S-LPS transfer from CD36 to TLR4/MD-2 (omitting CD14) is excluded by the observation that in both WT and CD36-/- PEMs cytokine production is strictly CD14-dependent (Fig 5D). In contrast, S-LPS induced strong binding of rCD14 to CD36 on PEMs also in the absence of serum (Fig 3D). When exposed to 20 ng/ml of monomeric S-LPS/rCD14 complexes, CD36-/- PEMs produced ~4-5-times less TNF-α and RANTES than WT PEMs (S6 Fig). These results indicate that under serum-free conditions, association of S-LPS/CD14 complexes with CD36 promotes S-LPS transfer from CD14 to TLR4/MD-2.

### CD36 positively regulates activation of TRIF-dependent TLR4 signaling by promoting internalization of LPS/TLR4/MD-2 complexes

**Fig 6:**
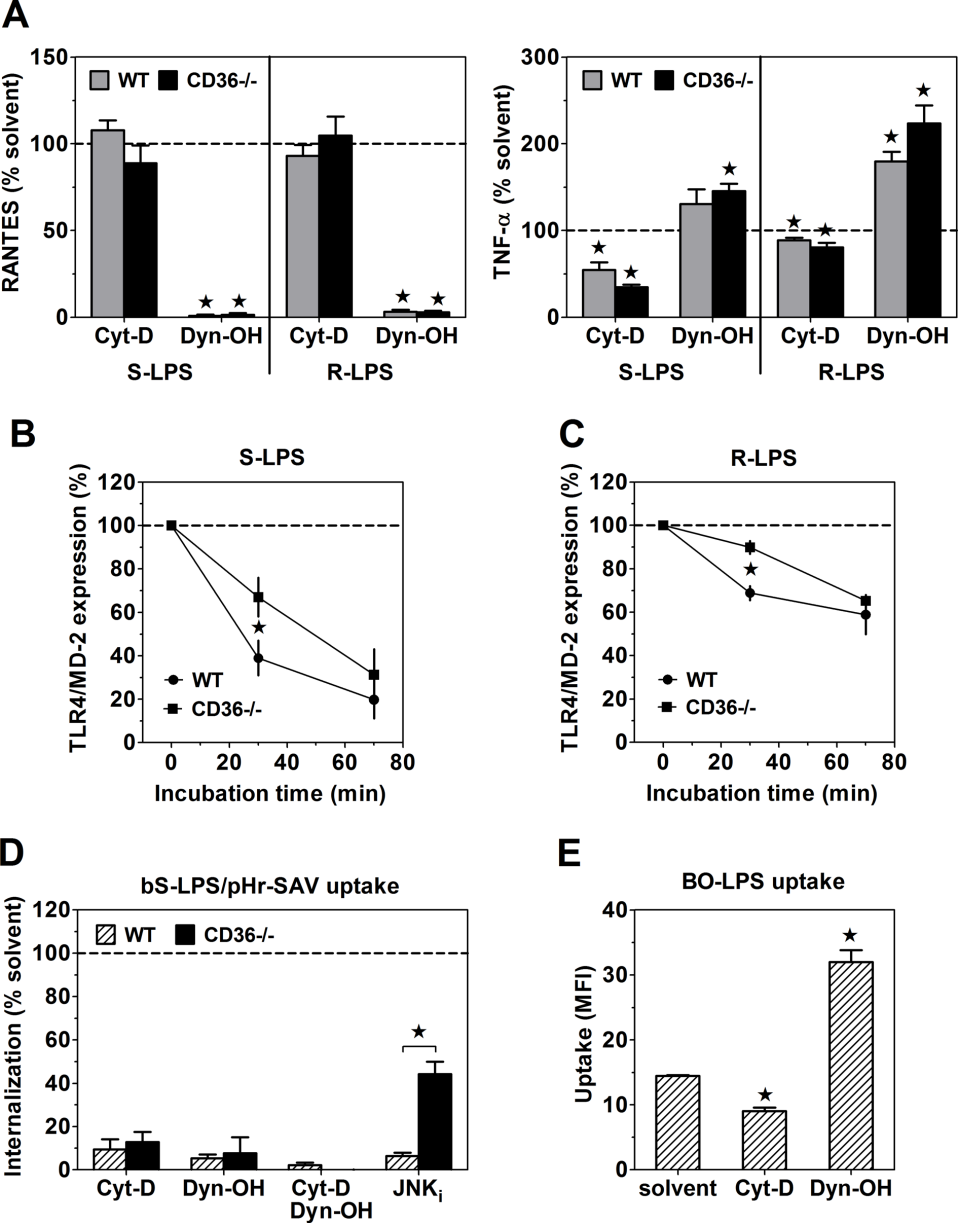
The role of endocytosis in LPS-induced intracellular signaling. (A) Hydroxy-dynasore (Dyn-OH) at 30 μM blocks RANTES, but enhances TNF-α production, stimulated by 1-h incubation on ice with 1 μg/ml LPS. Cytochalasin D (Cyt-D) at 10 μM has no effect on RANTES, but inhibits TNF-α production. (B, C) One μg/ml S-LPS (B) and R-LPS (C) produce stronger down-regulation of cell surface TLR4/MD-2 expression in WT than in CD36-/- PEMs. (D) Internalization of CD14-bound bS-LPS/pHr-SAV complexes into acidic endosomes is blocked by both Dyn-OH and Cyt-D. A JNK inhibitor (JNKi) at 20 μM produces stronger inhibition of the internalization in WT than in CD36-/- PEMs. (E) Seventy-min uptake of 10 μg/ml BO-LPS by WT PEMs is inhibited by Cyt-D, but enhanced by Dyn-OH. Graphs show means +SEM from 3–4 independent experiments, each performed in 3–4 replicates. *, significant inhibition (significantly different (p < 0.05) from 100 in one-sample t-test) (A), significant difference between WT and CD36-/- PEMs (p < 0.05 in two-tailed t-test) (B, C, D), or significantly different from the solvent control (p < 0.05 in ANOVA, followed by the Dunnett’s post-test) (E).

Unlike in experiments involving pre-incubation on ice (Fig 4B), 40-min pre-incubation with 200 ng/ml S-LPS at 37°C stimulated similar RANTES production in WT and CD36-/- PEMs (Fig 5C). These results might indicate that under conditions enabling endocytosis, decreased stimulation of cell surface-localized TLR4/MD-2 in WT PEMs is compensated by CD36-mediated delivery of S-LPS/CD14 complexes into endosomes and activation of the TRIF-dependent pathway by intracellular TLR4/MD-2 [38, 39]. However, this possibility is inconsistent with the observation that blocking of cell surface-localized TLR4/MD-2 with MTS-510 mAb inhibited S-LPS-stimulated RANTES production to a similar extent in WT and CD36-/- PEMs (S7A Fig). Moreover, S-LPS-induced RATES production was completely blocked by dynamin inhibition with hydroxy-dynasore (Fig 6A), which prevents activation of TRIF-dependent signaling by cell surface-derived TLR4/MD-2 complexes, but has little or no effect on the recruitment to endosomes/phagosomes and activation of intracellular TLR4/MD-2 [38]. Collectively, these results indicate the exclusive role of cell surface-derived TLR4/MD-2 in the stimulation of RANTES production by S-LPS.

Alternatively, CD36 might be involved in the internalization of LPS/TLR4/MD-2 complexes, which is required for the initiation of TRIF-dependent signaling. This possibility has been confirmed by results shown in Fig 6B. Thirty-min incubation with 1 μg/ml S-LPS produced ~1.8-fold stronger down-regulation of TLR4/MD-2 expression on the surface of WT (by ~61%) relative to CD36-/- PEMs (~33%).

**Fig 7:**
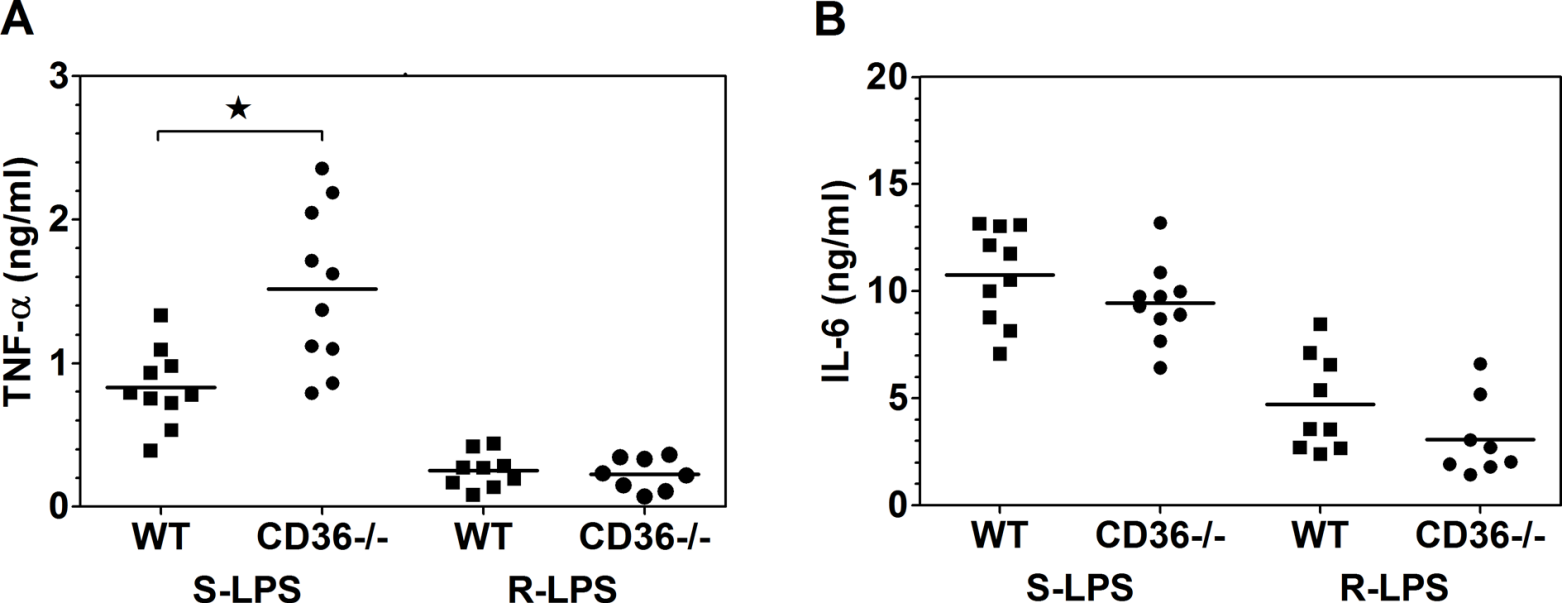
S-LPS stimulates significantly higher serum levels of TNF-α in CD36-/- than in WT mice. Mice were *i*.*p*. injected with 1 μg S-LPS or R-LPS in 1 ml BSA-PBS and 90 min later sera were collected for TNF-α and IL-6 determination by ELISA. Points represent cytokine concentrations in individual mice and horizontal lines mean values. *, p < 0.05 in two-tailed t-test.

To assess the role of CD36 in regulating S-LPS-induced responses in vivo, WT and CD36-/- mice were injected with 1 μg S-LPS *i*.*p*. and 90 min later sera were collected for cytokine determination. S-LPS induced on average ~1.8-fold higher serum levels of TNF-α in CD36-/- than in WT mice (Fig 7A), indicating that the S-LPS-induced, MyD88-dependent TLR4 signaling is subjected to the CD36-mediated negative regulation also in vivo. In contrast, serum levels of IL-6, which production under these experimental conditions is more TRIF- than MyD88-dependent [40] were not significantly affected by CD36 deficiency (Fig 7B).

### Dynamin controls both internalization of CD14-bound S-LPS into acidic endosomes and activation of the TRIF-dependent pathway of TLR4 signaling

The mechanisms of LPS endocytosis following its binding to CD14 have not been completely elucidated. As CD14 is a GPI-anchored protein, LPS/CD14 complexes might be internalized through the clathrin- and dynamin-independent, lipid raft-mediated pathway which depends on Cdc42 GTPase-regulated polymerization of actin [41, 42]. In contrast, LPS/TLR4/MD-2 complexes have been reported to undergo dynamin-dependent internalization through clathrin-coated pits [35, 43], which does not depend on the actin cytoskeleton [41, 44]. However, actin has been reported to control the transfer of cargo internalized through both clathrin-dependent and -independent pathways from early to late endosomes [45, 46]. To explore how these steps might affect S-LPS interactions, we assessed effects of hydroxy-dynasore, a dynamin-selective inhibitor, and of cytochalasin D, an inhibitor of actin polymerization, on the internalization into acidic endosomes of bS-LPS/pHr-SAV complexes. Internalization of these complexes was blocked by both cytochalasin D and hydroxy-dynasore (Fig 6D). Pre-incubation with hydroxy-dynasore also blocked activation of TRIF-dependent signaling, as assessed by RANTES production, while concomitantly enhancing MyD88-dependent signaling from the cell surface, as reflected by increased TNF-α production (Fig 6A). In contrast, cytochalasin D had no effect on RANTES production, but inhibited TNF-α production (Fig 6A), much more strongly in response to S-LPS (~50%) than to R-LPS (~10%). These results are consistent with a scenario in which, following dynamin-dependent internalization, S-LPS induces the TRIF-dependent signaling in early endosomes and then undergoes actin-dependent transfer to late endosomes/lysosomes.

CD36-medited endocytosis has been reported to depend on the activation of JNK [47]. The JNK-selective inhibitor SP600125 produced significantly stronger inhibition of the internalization of bS-LPS/pHr-SAV complexes in WT (~94%) than in CD36-/- (~56%) PEMs (Fig 6D), suggesting that CD36-mediated internalization of these complexes also depends on JNK activity.

Uptake of BO-LPS aggregates in WT PEMs was inhibited by ~40% by cytochalasin D and enhanced ~2-fold by hydroxy-dynasore (Fig 6E). The enhancing effect of hydroxy-dynasore may indicate that blocking of clathrin-dependent endocytosis causes compensatory up-regulation of clathrin-independent pathways, which are involved in BO-LPS uptake, as has been observed previously [27]. On the other hand, an incomplete inhibition of BO-LPS uptake by cytochalasin D may result from the partial re-direction of BO-LPS uptake to the clathrin-mediated pathway [48].

### CD36 and CD14 independently mediate R-LPS loading onto TLR4/MD-2

Unlike in the case of S-LPS (Fig 4B), WT and CD36-/- PEMs produced similar amounts of TNF-α and RANTES upon pre-incubation with R-LPS (Fig 4D). However, effects of CD14 blockade have revealed differences between these strains of PEMs. Whereas anti-CD14 mAb inhibited TNF-α production in CD36-/- PEMs by half, it had no effect in WT and SR-A-/- PEMs (Fig 4C). Likewise, this mAb produced almost complete, ~87% inhibition of R-LPS-stimulated RANTES production in CD36-/- PEMs, but only less than 35% inhibition in WT or SR-A-/- PEMs (Fig 4C). The observation that anti-CD14 mAb inhibits R-LPS-stimulated TNF-α production only in CD36-/- PEMs indicates that in WT macrophages CD36 may replace CD14 in R-LPS loading onto TLR4/MD-2. The stronger effect of anti-CD14 mAb on R-LPS-stimulated RANTES than TNF-α production may be explained by the additional role played by CD14 in the activation of TRIF-dependent pathways [10–12].

In addition, stimulation with R-LPS at 37°C led to similar TNF-α production in WT and CD36-/- PEMs (Fig 5C), consistent with similar binding of R-LPS to TLR4/MD-2 in both strains of PEMs (Fig 5B). Likewise, CD36 deficiency had no effect on the production of TNF-α, stimulated by 1 μg of R-LPS in vivo (Fig 7A). Thus, in contrast to S-LPS, R-LPS binding to TLR4/MD-2 does not seem subject to CD36-mediated negative regulation, likely because R-LPS is ineffective in stimulating CD14 association with CD36 (Fig 3D). In contrast, activation of the TRIF pathway by R-LPS was severely impaired in CD36-/- PEMs, as indicated by ~44% lower production of RANTES (Fig 5C). Similar to the case of stimulation with S-LPS, this impairment may be explained by the role of CD36 in mediating internalization of R-LPS/TLR4/MD-2 complexes. R-LPS produced ~3-times weaker down-regulation of surface TLR4/MD-2 in CD36-/- (~10%) than in WT (~31%) PEMs (Fig 6C).

As LPS binding to CD14 is facilitated by LBP from serum and, conversely, serum decreases LPS binding to CD36, we postulated that the absence of serum would increase the role of CD36 as a R-LPS receptor. Indeed, under serum-free conditions CD36-/- PEMs exhibited severe impairment of R-LPS binding to TLR4/MD-2 (Fig 5B) as well as of R-LPS-stimulated TNF-α (~35% reduction) and RANTES (~40%) production (Fig 5C). Whereas anti-CD14 mAb abolished R-LPS-stimulated cytokine production in CD36-/- PEMs, it produced only partial inhibition in WT PEMs (Fig 5D). Thus, unlike in serum-containing medium, in serum-free medium the role of CD36 in R-LPS loading onto TLR4/MD-2 could not be fully compensated by CD14. The complete blockade of R-LPS-induced responses by anti-CD14 mAb in CD36-/- PEMs (Fig 5D) also indicates that CD36 and CD14 are the only receptors mediating R-LPS loading onto TLR4/MD-2. Moreover, the additivity of effects of CD36 deficiency and of anti-CD14 mAb indicates that CD36 and CD14 mediate R-LPS loading onto TLR4/MD-2 independently of each other.

### R-LPS and S-LPS differ in their affinities for LBP, CD14 and CD36

**Fig 8:**
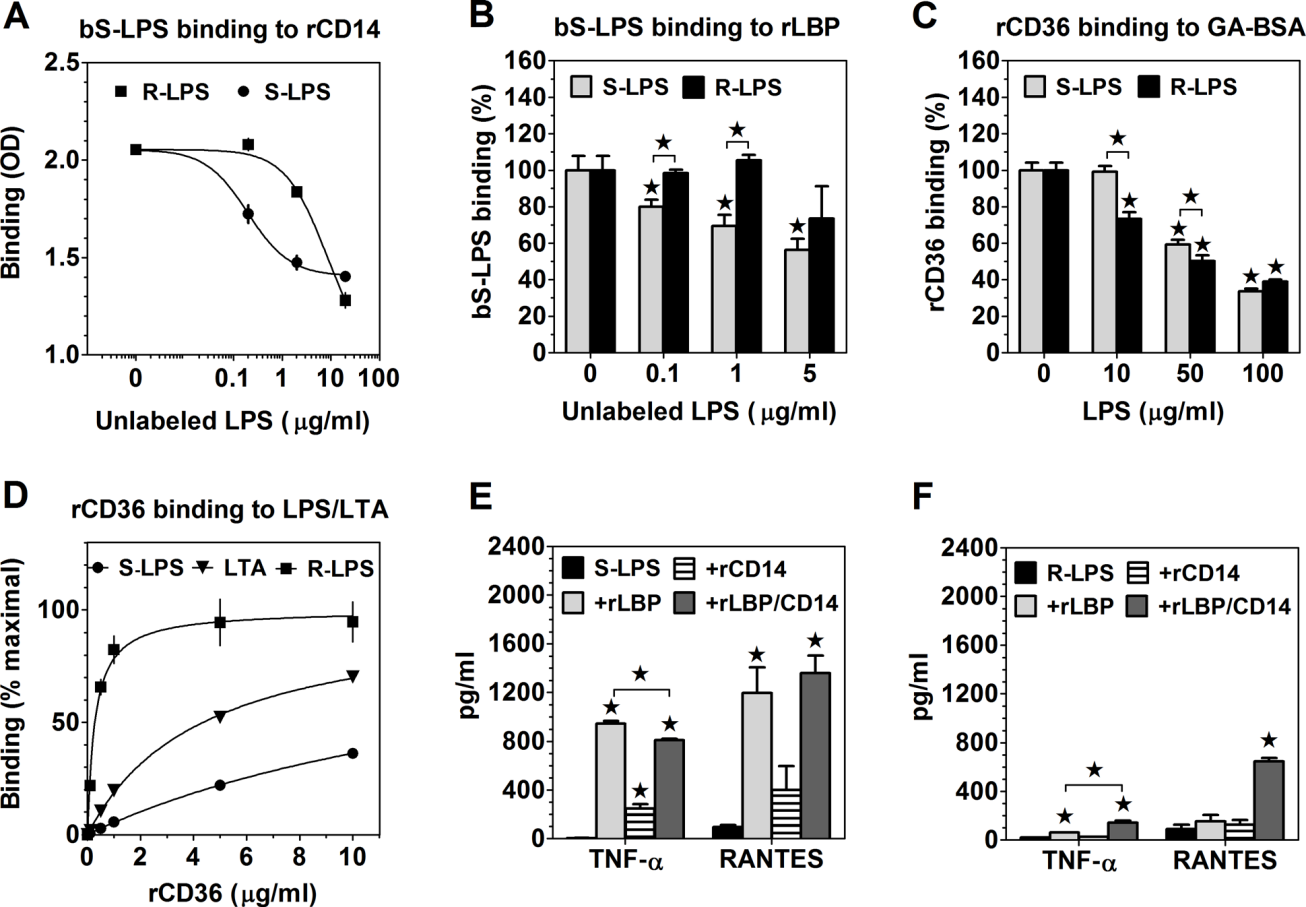
S-LPS and R-LPS bind to LBP, CD14 and CD36 with different affinities. (A) 200 ng/ml bS-LPS binding to plate-adsorbed rCD14 is more effectively inhibited by S-LPS than R-LPS. (B) S-LPS is a more efficient competitor than R-LPS in inhibiting bS-LPS binding to plate-adsorbed rLBP. (C) Lower concentrations of R-LPS than S-LPS are required to inhibit rCD36 binding to plate-adsorbed GA-BSA. (D) rCD36 binds with higher affinity to plate-adsorbed R-LPS than to S-LPS or LTA. (E) Cytokine production stimulated by 5 ng/ml S-LPS in serum-free medium is strongly enhanced by 0.2 μg/ml rLBP and, to a lesser extent, by 2 μg/ml rCD14. (F) Only the combination of rLBP and rCD14 enhances cytokine production in response to 5 ng/ml R-LPS. Graphs show means ± SEM of 3–4 replicates, obtained in single experiments, performed twice with similar results. Data were analyzed with two-tailed and one-sample t-test (B and C) or with ANOVA, followed by the Tukey-Kramer test (E and F); *, p < 0.05.

Differences in relative roles played by CD36 and CD14 in macrophage responses to R-LPS *versus* S-LPS may be explained by differences in binding affinities. As shown in Fig 8A, S-LPS competed with bS-LPS binding to rCD14 much more effectively than R-LPS, with IC_50_ values of ~0.2 and 2.6 μg/ml, respectively. Corresponding inhibition constant values, calculated on the basis of the K_D_ value of bS-LPS binding determined in saturation experiments (S2A Fig) and average molecular weights of S-LPS and R-LPS (15.8 kDa [49] and 4 kDa [50], respectively), were 3.8 ± 0.36 nM, in the case of S-LPS, and 650 ± 82.5 nM, in the case of R-LPS. Likewise, rLBP bound R-LPS with a lower affinity than S-LPS (Fig 8B). Whereas S-LPS produced significant inhibition of bS-LPS binding to rLBP already at 100 ng/ml, R-LPS had no effect even at 10-fold higher concentrations. These differences in affinities correlated with the ability of rCD14 or rLBP to sensitize PEMs to low concentrations of LPS in serum-free medium. Whereas TNF-α production stimulated by 5 ng/ml S-LPS was enhanced ~200-fold by rLBP and ~50-fold by rCD14 (Fig 8E), that stimulated by 5 ng/ml R-LPS was only increased ~3-times by rLBP and not significantly affected by rCD14 (Fig 8F). S-LPS- or R-LPS-stimulated RANTES production was regulated by rLBP and rCD14 in a similar manner as TNF-α production (Fig 8E and 8F). Interestingly, the combination of rLBP and rCD14 exerted a synergistic effect on cytokine production stimulated by R-LPS (Fig 8F), but did not produce stronger enhancement of S-LPS-stimulated cytokine production than rLBP alone (Fig 8E).

Conversely, R-LPS turned out to be a higher affinity ligand of CD36 than S-LPS, as indicated by relative potencies in inhibiting rCD36 binding to plate-adsorbed GA-BSA (Fig 8C). A higher affinity of rCD36 to R-LPS, as compared to S-LPS, has been confirmed in direct binding experiments (Fig 8D). rCD36 bound to plate-adsorbed R-LPS with ~60-times higher affinity (K_D_ = 0.28 ± 0.057 μg/ml, corresponding to 3.8 ± 0.78 nM) than to adsorbed S-LPS (K_D_ = 17.6 ± 1.25 μg/ml or 241 ± 17.1 nM). rCD36 binding to LTA, used as a positive control, occurred with an intermediate K_D_ of 4.4 ± 0.30 μg/ml (60 ± 4.1 nM).

## Discussion

Our experiments have revealed a complex role for CD36 in regulating macrophage responses to LPS, which depends on the chemotype of LPS, its concentration and the presence of serum. In serum-containing medium, CD36 contributes to the internalization of S-LPS/CD14 complexes, which competitively decreases S-LPS transfer from CD14 onto the cell surface-localized TLR4/MD-2 and, consequently, reduces activation of MyD88-dependent signaling. By contrast, under serum-free conditions, association of S-LPS/CD14 with CD36 might promote S-LPS transfer from CD14 to TLR4/MD-2 and activation of TLR4-induced intracellular signaling by increasing the probability of S-LPS/CD14 encounters with TLR4/MD-2. In macrophage membranes, diffusion of a fraction of CD36 is limited to linear confinement regions, which enhances the probability of collisions between receptors. De-polymerization of F-actin was found to markedly reduce the fraction of linearly moving CD36, leading to suppression of receptor clustering and of CD36-mediated signaling [51]. This postulated mechanism may be expected to play a significant role only under conditions when low density of S-LPS/CD14 complexes is the limited factor in the activation of TLR4/MD-2. Indeed, relative to WT PEMs, CD36-/- PEMs produced by ~72% less TNF-α in response to 20 ng/ml of complexes of monomeric S-LPS with rCD14, but only by ~11% less TNF-α in response to 10-times higher concentration of these complexes (S6 Fig). In addition, there was no difference in cytokine production between WT and CD36-/- PEMs when high concentrations of S-LPS were used for stimulation in serum-free medium (S7C Fig). Thus, CD36 may mediate a fine-tuning of macrophage responses to S-LPS. By increasing the sensitivity of macrophages to S-LPS, CD36 might enable detection of fewer bacteria and, consequently, generation of a faster response to infection. On the other hand, in later phases of infection, following leakage of serum to the infection site, the CD36-mediated negative regulation may prevent an excessive production of pro-inflammatory cytokines, thereby protecting from septic shock.

Of note, CD36-/- PEMs produced significantly more TNF-α than WT cells when subjected to a brief stimulation with S-LPS, but not when S-LPS was continuously present during the entire period of the 3.5-h culture (S7B Fig). The observation that in CD36-/- mice S-LPS induced significantly higher serum levels of TNF-α indicates that the former conditions may better mimic the situation in vivo, likely because operating in vivo re-distribution, clearance, neutralization and detoxification processes may lead to rapid decrease of LPS concentration/bioactivity.

In contrast, CD36 deficiency had no effect on S-LPS-induced activation of the TRIF-dependent pathway, which mediates the adjuvant effect of LPS on adaptive immunity [52], likely because the enhancement of TLR4 signaling caused by increased S-LPS binding to cell surface-localized TLR4/MD-2 had been countered by the impaired internalization of S-LPS/TLR4/MD-2 complexes. This positive role of CD36 in the regulation of TRIF-dependent signaling was more evident in the case of R-LPS, as R-LPS binding to TLR4/MD-2 was not subjected to the CD36-mediated negative regulation. Relative to WT PEMs, in CD36-/- PEMs internalization of TLR4/MD-2 caused by R-LPS was decreased by ~68%, and RANTES production was reduced by ~44%.

CD14 has been reported to mediate internalization of TLR4/MD-2 complexes independently of TLR4 signaling [10–12], but it has remained unclear how CD14 is linked to the endocytic machinery and intracellular signaling, as it lacks both transmembrane and intracellular domains. In this study, we have identified CD36 as a receptor which may provide a transmembrane link for CD14. We have found that CD36 is the only macrophage surface component that binds rCD14. Moreover, internalization of both S-LPS/CD14 and LPS/TLR4/MD-2 complexes are impaired in CD36-/- macrophages. The role of CD36 as a transmembrane link for CD14 is further supported by observations that the Syk kinase, the phospholipase Cγ2 and an immunoreceptor tyrosine-based activation motif (ITAM)-containing adaptor proteins are involved in both CD14-dependent internalization of TLR4/MD-2 complexes [11, 12] and in CD36-mediated endocytosis [53] and that complexes of CD36 with its ligands undergo actin-dependent internalization into endosomal structures in which CD36 co-localizes with GPI-anchored proteins [47, 54].

The detailed mechanisms of CD36 involvement in both S-LPS/CD14 and LPS/TLR4/MD-2 endocytosis remain to be elucidated. As pHr exhibits bright fluorescence only in strongly acidic pH, with the use of this dye we might preferentially measure the fraction of LPS uptake which is directed into late endosomes/lysosomes. The complete blockade of bS-LPS/pHr-SAV internalization by hydroxy-dynasore may indicate that, during this short incubation, only the portion of LPS internalized through clathrin-coated pits, including bS-LPS transferred from CD14 onto TLR4/MD-2, reaches lysosomes [35, 43]. Internalization of bS-LPS/pHr-SAV complexes was decreased by half in CD36-/- PEMs, indicating a positive regulatory role of CD36 in this process. In contrast, GPI-anchored proteins internalized through clathrin-independent, lipid raft-dependent pathways have been shown to be mainly recycled back to the plasma membrane [42, 46, 48, 55]. The observed kinetics of LPS uptake in monocytes-macrophages is consistent with the possibility that a large portion of endocytosed LPS is recycled. We have found that PEM-mediated clearance from the culture medium of unlabeled LPS within 40 min reaches the maximal value of ~50% of its initial concentration and is not further increased by extending the incubation up to 2 h [19]. In CD14-transfected monocytic THP-1 cells internalization of membrane CD14-bound tritiated LPS reached plateau within 30–60 min, when only ~55% of these complexes was internalized [56]. CD36 seems to be also involved in this clathrin-independent endocytosis of LPS by PEMs, as indicated by a significant impairment of BO-LPS uptake in CD36-/- PEMs, and by observed in our previous study strong inhibition exerted by anti-CD36 mAb on native LPS clearance from the culture medium by PEMs [19].

Our study has also revealed that, like CD14, CD36 differently regulates macrophage responses to S-LPS *versus* R-LPS and that these two receptors play complementary roles. Most importantly, it clarifies previously unexplained observations that activation of MyD88-dependent pathways of TLR4 signaling by S-LPS, but not by R-LPS exhibits strict CD14-dependence. The apparent CD14-independence of macrophage activation by R-LPS results from the ability of CD36 to substitute for CD14 in R-LPS loading onto TLR4/MD-2. CD14 and CD36 seem to mediate R-LPS loading onto TLR4/MD-2 independently of each other. This contrast with the mechanism of the facilitating effect of CD36 on S-LPS binding to TLR4/MD-2 in serum-free medium, a process which is entirely CD14-dependent. Moreover, the blockade of R-LPS-stimulated responses in CD36-/- PEMs by anti-CD14 mAb in serum-free (Fig 5D) and almost complete inhibition in serum-containing medium (S7D Fig) indicate that CD36 and CD14 may be the only accessory receptors mediating R-LPS loading onto TLR4/MD-2.

Differential involvement of LBP, CD14 and CD36 in macrophage activation by R-LPS *versus* S-LPS might be explained by the large differences in binding affinities found in our study. Applying a different approach, we have confirmed the previously suggested mechanism of LBP and CD14 cooperation in LPS loading onto TLR4/MD-2, involving LBP-catalyzed extraction of monomers from LPS micelles and their transfer to CD14 [8]. Our novel finding is that S-LPS transfer between LBP and CD14 is reversible, which is made possible by similar affinities of S-LPS for both proteins. rLBP and rCD14 bound S-LPS with much higher affinities than R-LPS. LPS binding to CD14 seems to be mainly mediated by the hydrophobic cavity present in its N-terminal part, which may accommodate some of acyl chains of lipid A [57]. The higher affinity of S-LPS for CD14 might result from the fact that, unlike in the case of R-LPS, S-LPS binding to CD14 additionally involves binding of the O-antigen to the grooves present outside this hydrophobic pocket [57, 58]. In addition to differences in binding affinities, differences in the structure of micelles formed by R-LPS and S-LPS might be partially responsible for the observed differences in the effectiveness of LBP and CD14 in functioning as accessory proteins for different LPS chemotypes. S-LPS may be much more available for receptors compared to R-LPS because the hydrophilic O-antigen can act as a water-solubilizing carrier for lipid A, which might explain the observation that despite the fact that commercial preparations of nominally smooth LPS, as this used in our study, usually contain variable amounts of R-LPS [59], effects of contaminating R-LPS in such S-LPS preparations are never apparent. Consistent with this interpretation, rLBP alone was sufficient to produce the maximal enhancement of macrophage responses to S-LPS, but the combined action of rLBP and rCD14 was required to produce disaggregation of R-LPS.

Conversely, rCD36 bound R-LPS with higher affinity than S-LPS. An essential property of SR ligands is their net negative charge. Negative charge is conferred on LPS molecules by phosphate groups, which esterify positions 1 and 4’ of the diglucosamine backbone of lipid A and some heptulose or KDO residues in the inner core oligosaccharide, and by carboxylic groups of KDOs [50]. Thus, the lower affinity of S-LPS for CD36, relative to R-LPS, may result from lower density of negative charges in S-LPS micelles, caused by the attachment of uncharged O-antigens, which account for as much as 67–78% of the total molecular weight of S-LPS [59]. Our findings that relative to S-LPS, R-LPS exhibits lower affinity for CD14, but higher affinity for CD36 may explain previous observations that with the reduction of the size of its saccharide part, the potency of LPS to activate CD14+/+ macrophages decreases, but responses of CD14-/- macrophages are enhanced [60].

In order to be able to bind to SR-A with high affinity, ligands need to have an even stronger negative charge than that sufficient to confer a high-affinity binding to CD36 [34, 61]. Thus, concentration of negative charges associated with phosphorylated diglucosamine and the inner core oligosaccharide on the surface of lipid A or Re-LPS micelles may enable their binding to SR-A [20–22]. In contrast, masking of these negative charges by the attached uncharged saccharides of the outer core and the O-antigen in S-LPS micelles seems to prevent their binding to SR-A, as we have found that SR-A does not serve as either a binding or a signaling receptor for S-LPS. Binding to SR-A of fluorescent S-LPS conjugates, observed in other studies [25–27], was likely mediated by the attached, negatively-charged fluorochromes rather than by S-LPS itself.

Our studies have identified surprising complexity in the interaction of LPS chemotypes with macrophage receptors and signal transduction machinery. The data explain prior confounding results, and also suggest a biologic basis for variability in the timing and strength of macrophage activation at infection sites that differ in availability of serum and in LPS chemotypes released.

## Supporting Information

S1 Fig Binding of bS-LPS (A, B) and rCD14 (C) to PEMs. (A) CD36 and SR-A deficiencies have no effect on 1-h bS-LPS biding at 37°C to metabolically-poisoned PEMs in either serum-free or serum-containing medium. (B) Binding of 200 ng/ml bS-LPS to WT PEMs on ice is blocked by a 200-fold excess of unlabeled S-LPS. (C) 1 μg/ml S-LPS stimulates higher rCD14 binding to PEMs at 0°C than at 37°C.
(TIF)

S2 Fig bS-LPS binding to recombinant receptors. (A) Binding of bS-LPS to plate-adsorbed rCD14 is much higher than that to rCD36. Serum strongly increases level of bS-LPS binding to rCD14 without altering its affinity. (B) The kinetics of bS-LPS binding to rCD14 is much slower in BSA-PBS than in FCS-PBS. (C) The inclusion of 200 ng/ml rLBP in BSA-PBS increases bS-LPS binding to rCD14. (D) bS-LPS binds dose-dependently to rLBP. (E) 200 ng/ml rLBP produces already the maximal acceleration of bS-LPS binding to rCD14.
(TIF)

S3 Fig **rCD36 binds bS-LPS specifically, but does not facilitate bS-LPS binding to rCD14** (A) Anti-CD36 mAb inhibits bS-LPS binding to rCD36 in BSA-PBS. (B) rCD36 and rCD14 exhibit similar adsorption to plates. (C) Soluble rCD36 has no effect on bS-LPS binding to adsorbed rCD14 in BSA-PBS.
(TIF)

S4 Fig CD36 contributes to S-LPS uptake by PEMs, whereas the binding of fluorescent S-LPS conjugates to SR-A is mediated by fluorophores. (A) Both CD36- and SR-A-deficient PEMs exhibit significant impairment of 70-min AF-LPS uptake. (B) Uptake of 1 μg/ml AF-LPS by PEMs is strongly inhibited by 100 μg/ml DS, but unaffected by 250-fold excess of unlabeled S-LPS. (C) Metabolic poisoning blocks internalization of pHr-labeled, HOCl-oxidized ovalbumin (pHr-OVA-Cl) into acidic endosomal compartments. (D) Two-h uptake of AF-LPS by PEMs is not saturable. (E) Binding and uptake of BO-LPS is significantly decreased in CD36-/-, but not in SR-A-/- PEMs. (F) Unlabeled S-LPS only partially inhibits BO-LPS uptake by PEMs. Graphs A-B and E-F show means +SEM from 4–7 independent experiments. Graphs C and D show means +SEM of 4 replicates in a single experiment, which was performed twice with similar results. Data were analyzed with the repeated measures ANOVA, followed by the Dunnett’s post-test. *, p < 0.05; MFI, mean fluorescence intensity; ND, not done.
(TIF)

S5 Fig WT, CD36-/- and SR-A-/- PEMs express TLR4/MD-2 and CD14 at similar levels. (A) Expression of the indicated receptors on WT and SR-A-/- PEMs was determined by cellular ELISA. Amounts of HRP-conjugated, secondary Abs bound to cells were read from standard curves and specific binding calculated by subtracting binding of isotype-matched control mAb from the total binding of receptor-specific mAb. (B) Expression of receptors on CD36-/- PEMs was assessed by cellular ELISA and expressed as % of expression in WT PEMs. The data shown are averages +SEM from the indicated number (N) of independent experiments.
(TIF)

S6 Fig CD36-/- PEMs exhibit impaired cytokine production in response to low concentrations of pre-formed monomeric S-LPS/rCD14 complexes in serum-free medium. (TIF)

S7 Fig Roles of CD36, CD14 and TLR4/MD-2 in LPS-stimulated cytokine production by PEMs. (A) In serum-containing medium, RANTES production stimulated by 40-min incubation with 20 ng/ml S-LPS is inhibited by anti-TLR4/MD-2 MTS510 mAb to a similar extent in WT and CD36-/- PEMs. (B) Continuous, 3.5-h stimulation with S-LPS induces similar TNF-α production in WT and CD36-/- PEMs. The data shown are means +/- SEM from 7 independent experiments. (C) In serum-free medium, CD36-/- PEMs produce less cytokines than WT controls in response to low, but not high concentrations of S-LPS (D) Cytokine production, stimulated by 40-min incubation with R-LPS in FCS-RPMI is inhibited by anti-CD14 mAb more strongly in CD36-/- than WT PEMs.
(TIF)
