# Supplementary figures and images for: CD36 Differently Regulates Macrophage Responses to Smooth and Rough Lipopolysaccharide
Source: PLoS One. 2016 Apr 13;11(4):e0153558. doi: 10.1371/journal.pone.0153558 (PMC4830570; doi:10.1371/journal.pone.0153558)

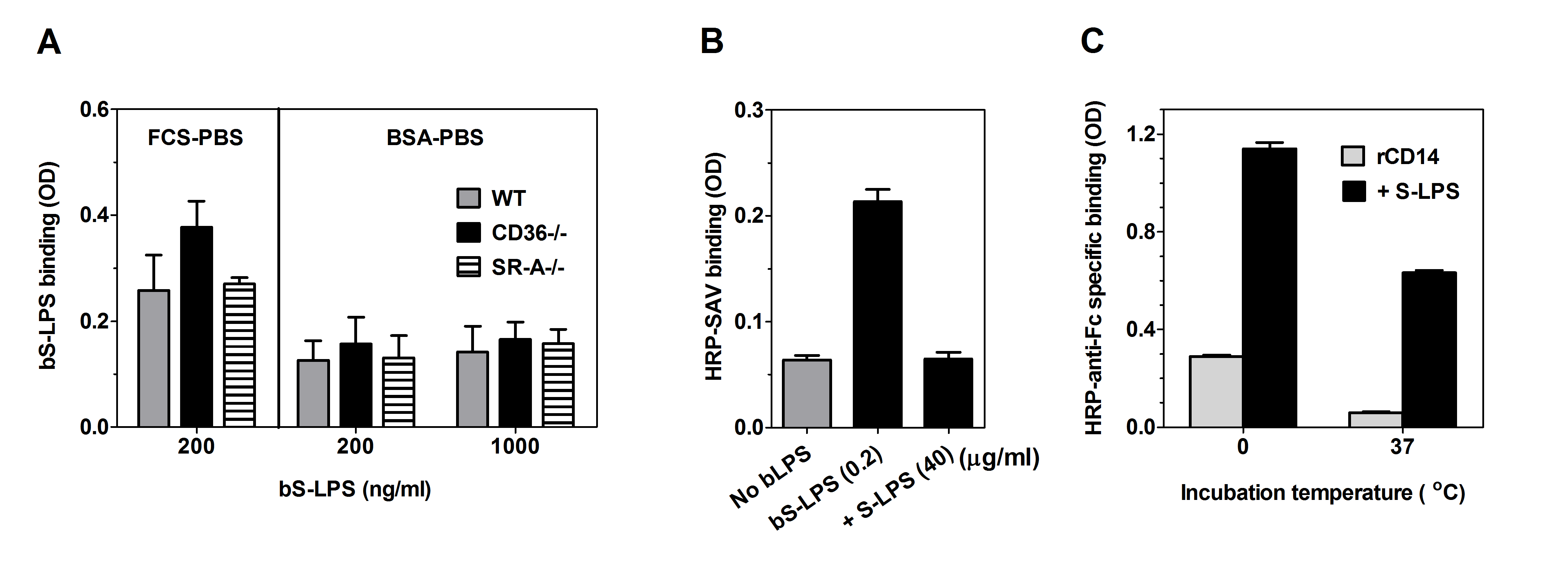

Supplement: S1 Fig — (A) CD36 and SR-A deficiencies have no effect on 1-h bS-LPS biding at 37°C to metabolically-poisoned PEMs in either serum-free or serum-containing medium. (B) Binding of 200 ng/ml bS-LPS to WT PEMs on ice is blocked by a 200-fold excess of unlabeled S-LPS. (C) 1 μg/ml S-LPS stimulates higher rCD14 binding to PEMs at 0°C than at 37°C. (TIF) [file pone.0153558.s001.tif]

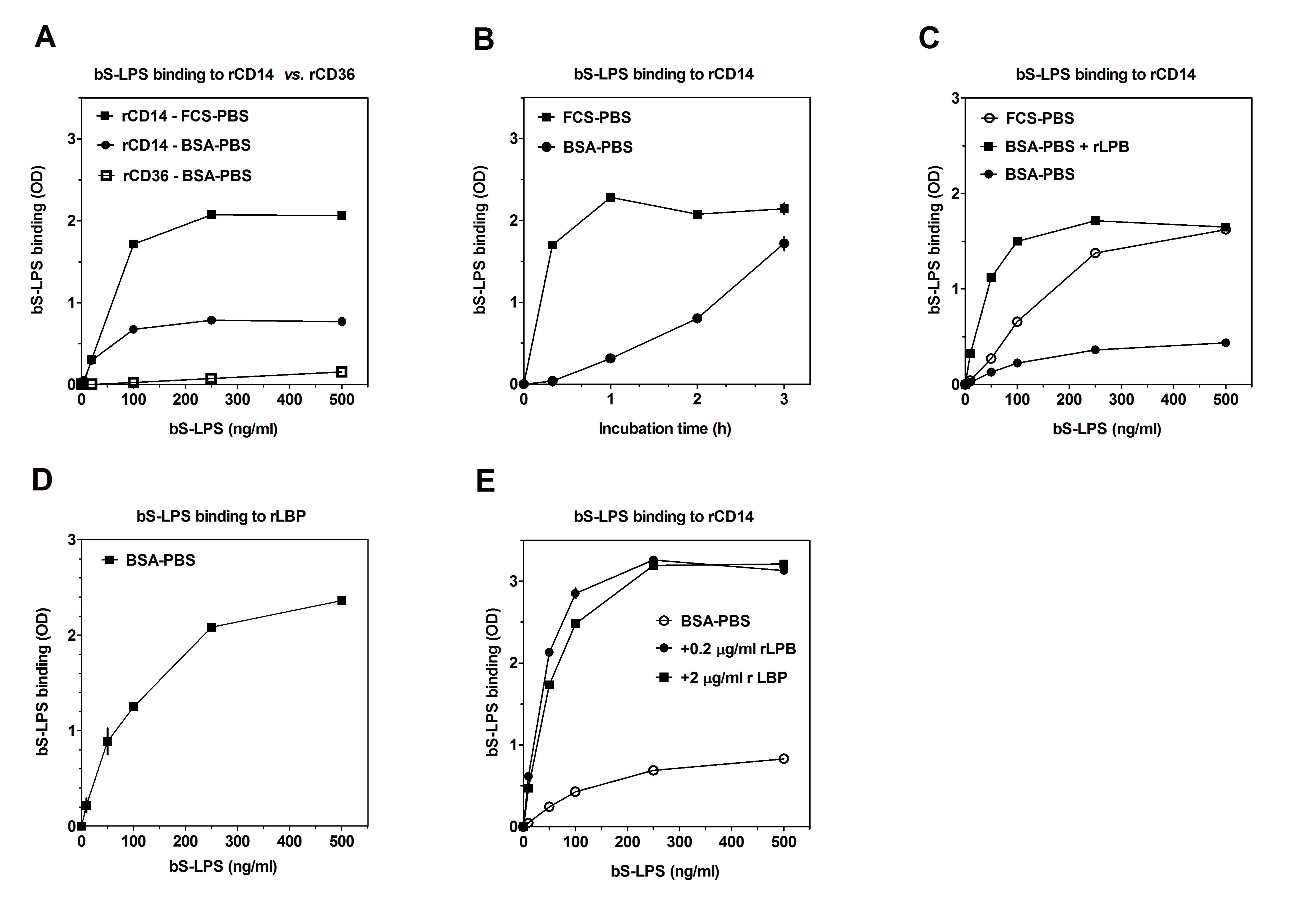

Supplement: S2 Fig — (A) Binding of bS-LPS to plate-adsorbed rCD14 is much higher than that to rCD36. Serum strongly increases level of bS-LPS binding to rCD14 without altering its affinity. (B) The kinetics of bS-LPS binding to rCD14 is much slower in BSA-PBS than in FCS-PBS. (C) The inclusion of 200 ng/ml rLBP in BSA-PBS increases bS-LPS binding to rCD14. (D) bS-LPS binds dose-dependently to rLBP. (E) 200 ng/ml rLBP produces already the maximal acceleration of bS-LPS binding to rCD14. (TIF) [file pone.0153558.s002.tif]

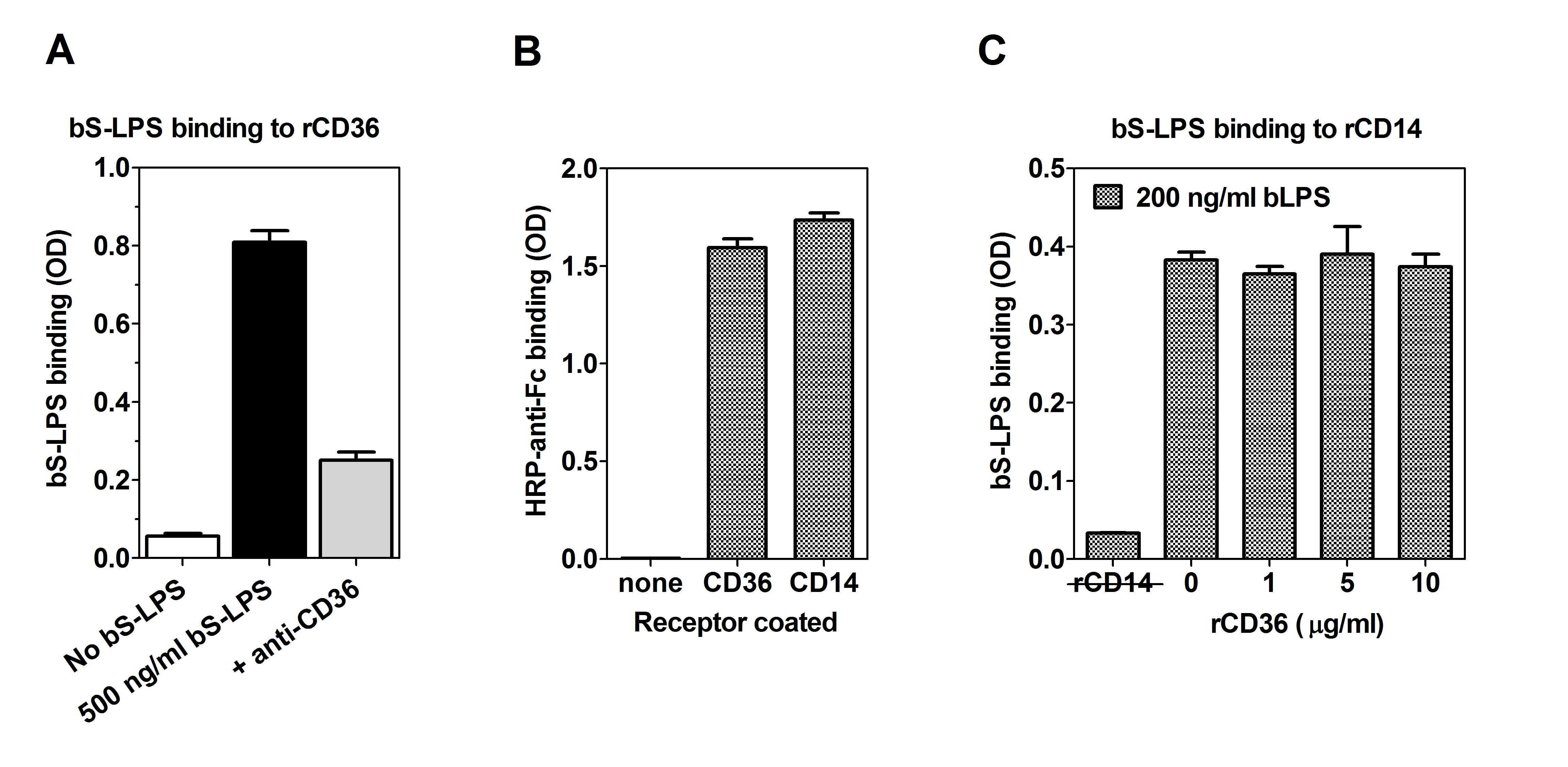

Supplement: S3 Fig — rCD36 binds bS-LPS specifically, but does not facilitate bS-LPS binding to rCD14 (A) Anti-CD36 mAb inhibits bS-LPS binding to rCD36 in BSA-PBS. (B) rCD36 and rCD14 exhibit similar adsorption to plates. (C) Soluble rCD36 has no effect on bS-LPS binding to adsorbed rCD14 in BSA-PBS. (TIF) [file pone.0153558.s003.tif]

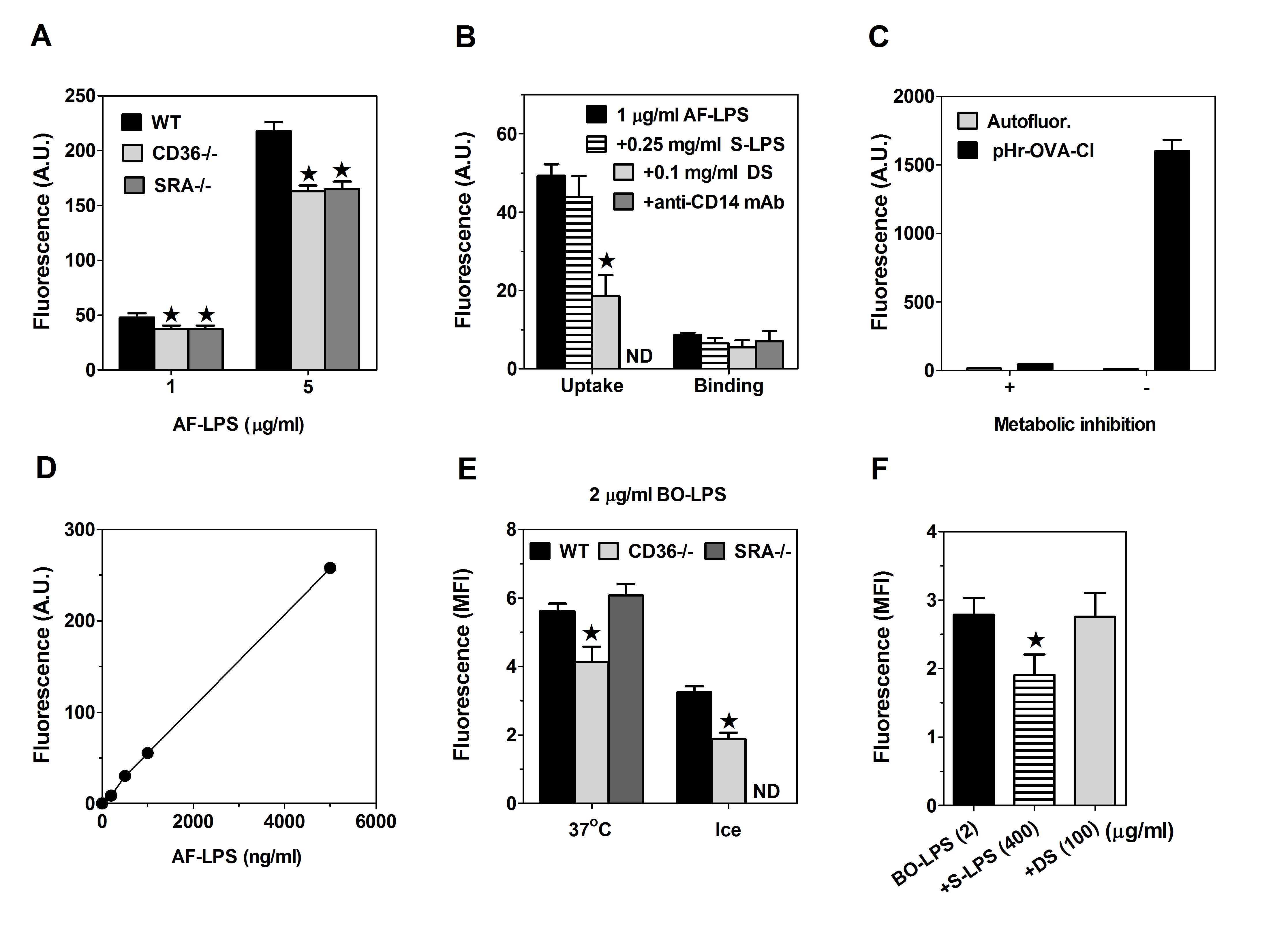

Supplement: S4 Fig — (A) Both CD36- and SR-A-deficient PEMs exhibit significant impairment of 70-min AF-LPS uptake. (B) Uptake of 1 μg/ml AF-LPS by PEMs is strongly inhibited by 100 μg/ml DS, but unaffected by 250-fold excess of unlabeled S-LPS. (C) Metabolic poisoning blocks internalization of pHr-labeled, HOCl-oxidized ovalbumin (pHr-OVA-Cl) into acidic endosomal compartments. (D) Two-h uptake of AF-LPS by PEMs is not saturable. (E) Binding and uptake of BO-LPS is significantly decreased in CD36-/-, but not in SR-A-/- PEMs. (F) Unlabeled S-LPS only partially inhibits BO-LPS uptake by PEMs. Graphs A-B and E-F show means +SEM from 4–7 independent experiments. Graphs C and D show means +SEM of 4 replicates in a single experiment, which was performed twice with similar results. Data were analyzed with the repeated measures ANOVA, followed by the Dunnett’s post-test. *, p < 0.05; MFI, mean fluorescence intensity; ND, not done. (TIF) [file pone.0153558.s004.tif]

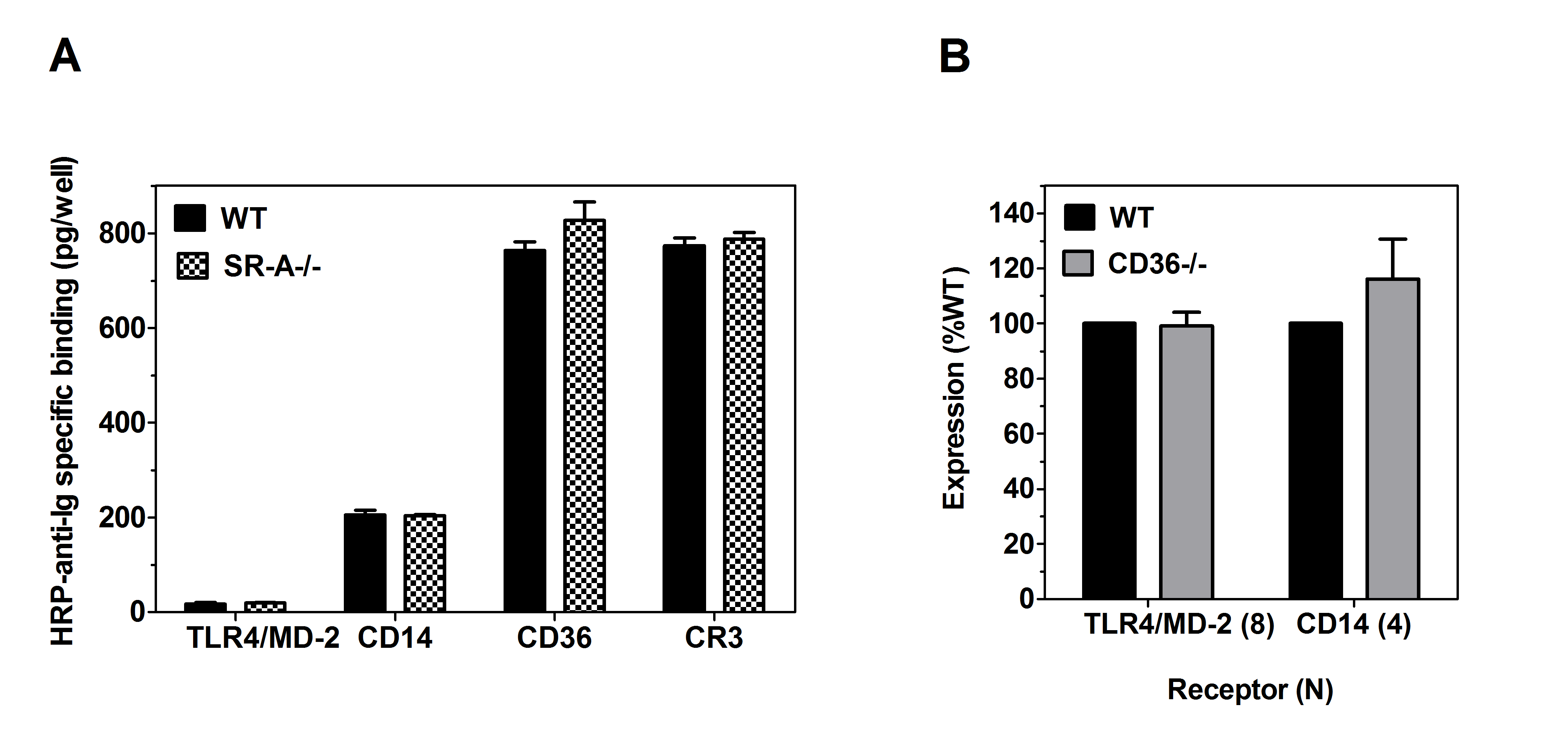

Supplement: S5 Fig — (A) Expression of the indicated receptors on WT and SR-A-/- PEMs was determined by cellular ELISA. Amounts of HRP-conjugated, secondary Abs bound to cells were read from standard curves and specific binding calculated by subtracting binding of isotype-matched control mAb from the total binding of receptor-specific mAb. (B) Expression of receptors on CD36-/- PEMs was assessed by cellular ELISA and expressed as % of expression in WT PEMs. The data shown are averages +SEM from the indicated number (N) of independent experiments. (TIF) [file pone.0153558.s005.tif]

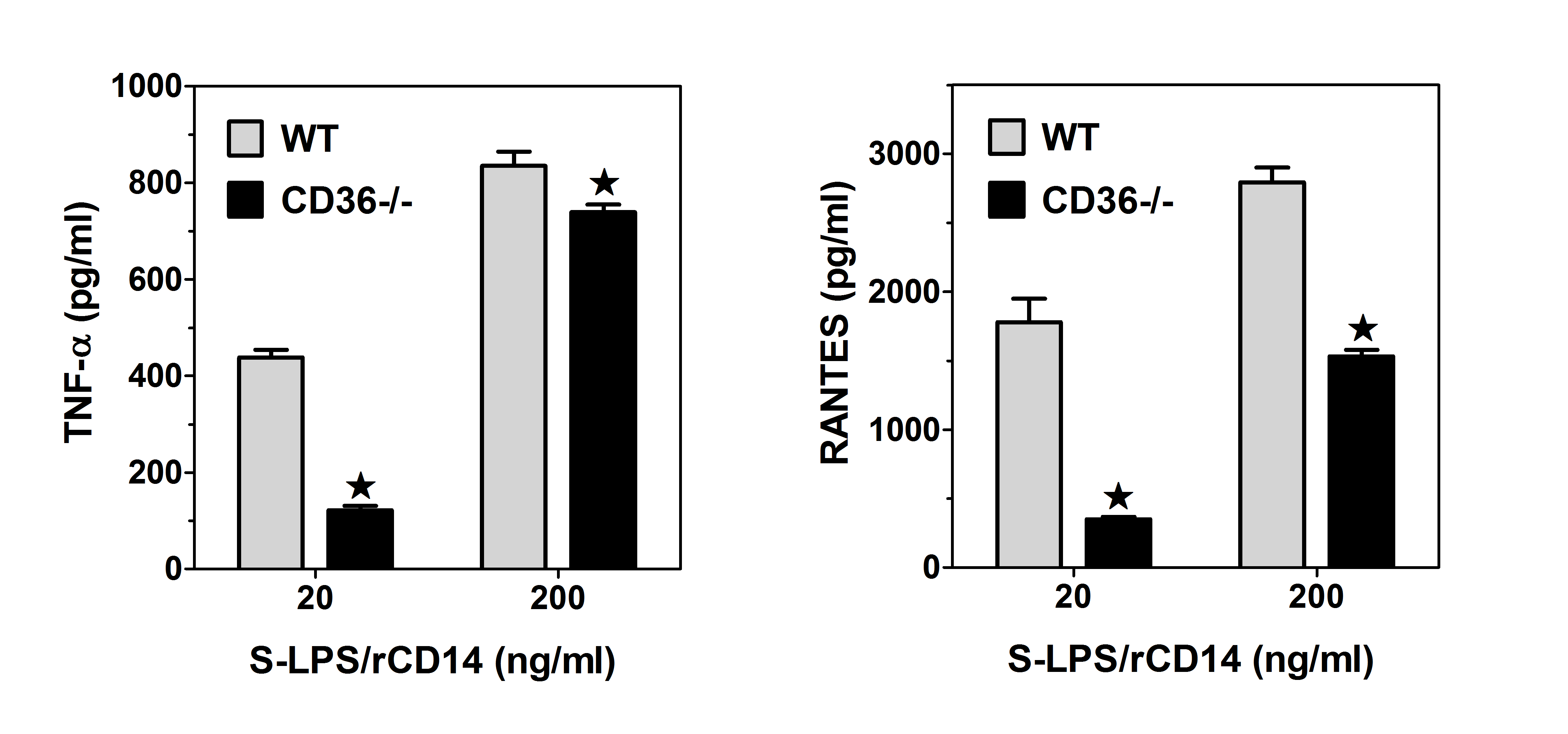

Supplement: S6 Fig — (TIF) [file pone.0153558.s006.tif]

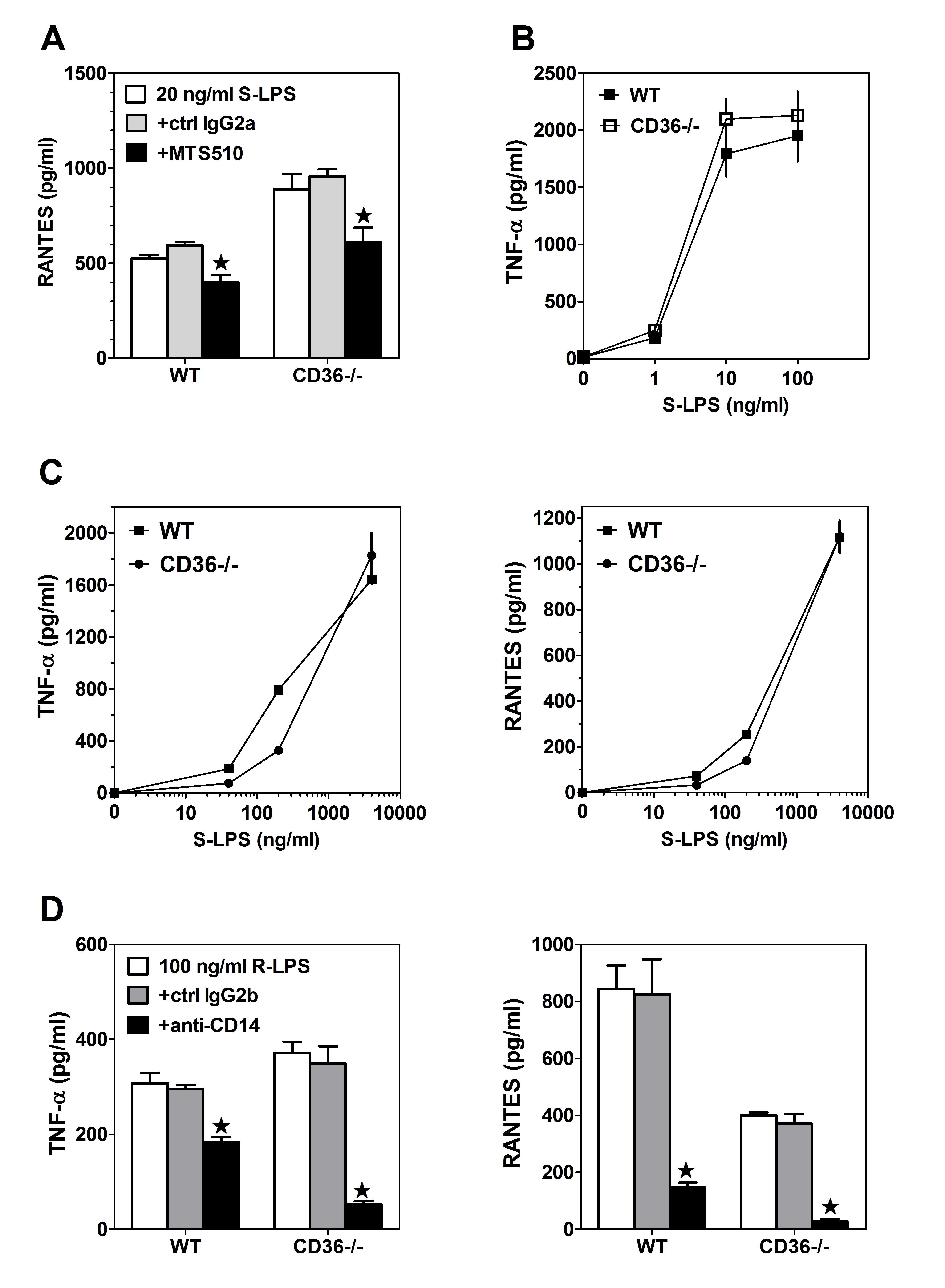

Supplement: S7 Fig — (A) In serum-containing medium, RANTES production stimulated by 40-min incubation with 20 ng/ml S-LPS is inhibited by anti-TLR4/MD-2 MTS510 mAb to a similar extent in WT and CD36-/- PEMs. (B) Continuous, 3.5-h stimulation with S-LPS induces similar TNF-α production in WT and CD36-/- PEMs. The data shown are means +/- SEM from 7 independent experiments. (C) In serum-free medium, CD36-/- PEMs produce less cytokines than WT controls in response to low, but not high concentrations of S-LPS (D) Cytokine production, stimulated by 40-min incubation with R-LPS in FCS-RPMI is inhibited by anti-CD14 mAb more strongly in CD36-/- than WT PEMs. (TIF) [file pone.0153558.s007.tif]
